# Experimental performance examination of a coherence technique-based numerical differential current relay for AC machine stator windings protection

**DOI:** 10.1038/s41598-025-89092-z

**Published:** 2025-03-05

**Authors:** R. A. Mahmoud

**Affiliations:** Department of Electrical Power and Machines Engineering (PME), College of Engineering Science & Technology, Misr University for Science and Technology (MUST), 6th of October City, Giza Egypt

**Keywords:** AC machine, Electrical faults, Inter-turn faults, Fault classification, Differential current relay, Coherence criterion, Energy science and technology, Engineering

## Abstract

A computational technique based on a coherence method for fault detection and classification for electrical machine stator windings is presented in this article. The coherence algorithm can identify clearly and concisely inter-turn and shunt faults situated on the 3-phase stator windings of the AC machine. Besides, it can categorize the different types of internal shunt faults**.** The cross-coherence algorithm performs the functional role of digital differential current to find and classify the internal faults; while, the auto-coherence algorithm acts as an overcurrent detector to define the occurrence of external, internal, or inter-turn faults. A new setup of three-phase induction machine stator windings, where each winding is re-winded to produce 20 taps per phase, is used to examine the approach. The new design is intended to build current transformers at the neutral and supply sides of the three windings, and to simplify conducting comprehensive examinations to verify the efficacy and efficiency of the advanced algorithm. The protection characteristics of the developed algorithm will be analyzed and estimated using the new setup. The test results indicate that the reliability and accuracy of the protection are above 98.7%. The coherence criterion is also useful for monitoring electrical faults, sensing inter-turn faults, distinguishing between external and internal shunt faults, classifying diverse internal shunt faults within the equipment protection zone, and estimating the tripping time when inter-turn faults occur. Furthermore, a new design of protection tripping-characteristic curves is established, and the time response of the computational technique is fast.

## Introduction

Induction machines remain a crucial component of electrical power systems. Due to their numerous applications, they may be prone to various electrical, magnetic, mechanical and thermal stresses, which may require fault diagnosis and machine maintenance^1^. It is important to develop protection methods that allow industry to find and precisely analyze various machine problems. The creation of pragmatic and microprocessor-based systems for fault analysis and the development of comprehensive techniques enable users to select the appropriate scheme for fault diagnosis^2^. In^3^, the authors proposed a method that uses Convolutional Neural Networks (CNNs) and current signature analysis to identify machine faults. Different faults detection in machine windings was achieved by using the CNNs algorithm, which automatically reduces relevant features from the measurements of current. In^4^, the method employed a combination of machine learning and wavelet transform to find and analyze faults. The wavelet transform was used to extract features from machine measurements, while machine learning was used to classify faults, which improved method’s accuracy. In^5^, transfer learning was integrated with few-shot learning for induction machines fault diagnosis. The approach enabled their module to be quickly adjusted to diverse fault kinds with less extra training data. Deep learning (DL) models for diagnosing motor faults were presented in^6^. The robustness and effectiveness of the method was increased by incorporating the outputs of many deep learning (DL) models. In^7^, wavelet packet decomposition for feature extraction was integrated with machine learning for machine fault diagnosis. The method extracted frequency information in details, which improved the accuracy of the technique for a classification of faults. In^8^, the approach used edge computing to detect faults in real-time for induction motors. The utilization of machine learning models on edge devices investigated to provide low-latency fault identification, which is advantageous for industrial applications. In^9^, a combination of Convolutional Neural Networks (CNN) and recurrent neural networks (RNN) was used to diagnose motor faults. The integrated model efficiently and effectively captured both spatial and temporal features from measured machine data.

Due to their high power rating and efficiency, Permanent Magnet Synchronous Motors (PMSMs) are vastly used in industrial fields^10^. The turn-to-turn faults of one machine stator winding may result in damaging impacts if these faults are not quickly defined and isolated^10^. Therefore, an online detection of turn-to-turn faults is indispensable to maintaining the machine stator windings. Many studies were conducted to diagnose the turn-to-turn faults of the AC machine windings. In^11^, the Fast Fourier Transform (FFT) was used to analyze current waveforms and detect this fault category. A low voltage signal at the motor standstill state supplies the PMSM excitation, and the resistance and inductance deduced from the current measurements can be used to quantify the number of short-turns and the fault location^12^. The square of the negative sequence components of the AC machine was used in^13^ as online fault indicators. In applications with high security requirements, a Dual‐Redundancy Permanent Magnet Synchronous Motor (DRPMSM) with no electromagnetic coupling and low thermal coupling can continue to work if any fault occurs on one of the two coils. It is possible to find turn-to-turn faults within the DRPMSM by differentiating between the direct axis voltage and reactive power^14,15^. However, the evaluation approach is sophisticated under different load currents, and measurements with constant sampling frequency are unable to diagnose the fault as the fault index fluctuates uncommonly at a low rotor speed. The auto-correlation was used in^16^ to detect only turn-to-turn faults located on the induction machine windings. It was unable to distinguish between external and internal faults, and it couldn’t classify the faults. In^17^, the magnetic pendulous oscillation technique was introduced to identify turn-to-turn faults in induction machines. The technique performance was analyzed using a dynamic forward model of the machine in the faulty and healthy states. The magnetic pendulous oscillation method used specified petals configuration to the relation between the current and voltage space vectors to determine the turn-to-turn faults^17^. A complex-vector dynamic model for induction machine stator windings in the state of turn-to-turn faults was described in^18^, where a substitutional approach of faulty leakage inductance was presented to estimate the leakage current. In^19^, the response of inverter switching harmonics in Common-Mode Voltage (CMV) was employed to discover the stator turn-to-turn faults in the inverter-fed induction machine in closed-loop control. In^20^, dual algorithms were used to diagnose off-line and on-line turn-to-turn faults for the stator windings of squirrel-cage induction machine. Machine parameters were estimated to find and pinpoint the faults; the first algorithm combined trust-region and Broyden–Fletcher–Goldfard–Shano methods to profit from their benefits; the second algorithm relied on the moving horizon estimation, and united off-line measurements and on-line parameters estimation with a large number of sampling rate to monitor in real time the stator turn-to-turn faults in induction machines. In^21^, a hybrid CNN–LSTM model for fault recognition in induction machines was proposed. The CNN-based technique was useful for feature extraction from the measured input signals, while the LSTM-based technique captured temporal dependencies, resulting in improved fault identification performance. A thermography-based AC machine fault detection using InceptionV3 model was proposed in^22^. Contrast Limited Adaptive Histogram Equalization (CLAHE) was applied to the input images for improving the protection accuracy, and the InceptionV3 could be integrated with a Squeeze-and-Excitation (SE) channel attention mechanism for enhancing the method performance^22^. A Domain Mixed-Enhanced Domain Generalization Network (DEMDGN) was applied to improve Imbalanced Fault Diagnosis (IFD) performance by using mixup-based data augmentation and domain-based difference metrics to align feature distributions across multiple heterogeneous source domains^23^. By establishing domain-invariant characteristics, DEMDGN was able to detect faults under diverse operational conditions^23^. Machine learning (ML) algorithm-based artificial neural network (ANN) and ANFIS (ANN and Fuzzy) models were used to detect inter-turn stator faults in induction machine-based pumping systems^24^. The analysis of the approach results was accomplished using a MATLAB Simulink model and a simulator based on loop (HIL)^24^.

Most conventional differential current protections used current phasors^25^. A crucial problem with the differential current relays (longitudinal type) originates when turn-to-turn faults occur, resulting in a reduced short-circuit turns^26^. The decreased number of turns included in the fault reduces circulating short-circuit currents (at both receiving and sending ends of the equipment) that blinds the conventional differential protection. The machine windings protection against grounding faults are protected with the restricted earth fault protection that has a higher sensitivity than the differential current protection^27,28^. However, the restricted earth fault can only be used for grounding and unsymmetrical faults, it is considered a secondary protection for the main differential protection, and more specific specifications for current transformers are taken into account^27,28^.

In^29^, the approach was relied on the alienation statistic derived by utilizing the coherence statistic for voltage and current measurements at generator load terminals. In^30^, the protection scheme used directly the coherence statistic for the voltage and current values. The fault detection time of the alienation-based statistic^29^ is therefore greater than that of the coherence-based statistic^30^. In^29,30^, the two algorithms are unable to (I) detect turn-to-turn faults, (II) differentiate between faults outside and inside the equipment protection zone, and (III) categorize the internal shunt faults that occur within the protection zone. To treat these issues, the proposed approach presents a reasonable solution based on the coherence concept. The methodology combines cross-coherence and auto-coherence techniques for fault diagnosis using only the current signals at two ends of the machine windings. The cross-coherence technique can fulfill the function of differential current protection to identify and classify the internal shunt faults; whereas, the auto-coherence technique can be used to work as an overcurrent protection to detect external, internal, and turn-to-turn faults. The method is experimentally tested on a three-phase induction machine. Its three-phase stator windings have been re-winded to obtain 20 taps per each phase to conduct comprehensive tests on the turns and windings of the machine.

The experimental findings confirm that the coherence-based method has the following benefits:

1. The fault instant can be determined using the coherence criterion,
2. Coherence trajectories can be constantly observed,
3. Turn-to-turn and shunt faults can be immediately found,
4. The approach can be applied to large-scale systems,
5. The technique develops a new design for quadratic tripping curves based on both auto-coherence and cross-coherence estimators, which is established to discriminate between turn-to-turn, internal, and external faults.
6. The method proposes other auto-coherence-inverse time curves, which can be used to estimate the operating time in the event of turn-to-turn faults,
7. The proposed algorithm can categorize the internal shunt faults within the AC machine protection region using the cross-coherence technique,
8. The approach can identify and assess the degree of imbalance and disturbance for the three-phase currents using the cross-coherence and auto-coherence coefficients, respectively.
9. The present methodology is fast, safe, dependable, reliable, and precise,
10. Single-phase or three-phase AC machines can be protected by the coherence criterion,
11. The functional roles of differential current, overcurrent, and current imbalance protections can be incorporated into the approach,
12. The approach is inactive when there are external faults and normal operations, whereas it is active in the events of an internal faults and turn-to-turn faults,
13. It is feasible to integrate the methodology with other microprocessor-based processes to enhance protection redundancy and reliability,
14. New disturbance and unbalance indicators can be developed using the coherence estimators for the three-phase current waveforms of the induction machine,
15. The protection attributes, such as speed, accuracy, selectivity, dependability, security, and reliability percentages, are superior,
16. The protection requirements do not rely on estimating prescribed settings to adjust restraining and tripping regions existing in the developed characteristic curves, and
17. The method can control protection speed, sensitivity, security, dependability, and stability.

This article is organized as follows: the methodology is explained with more description in Section “Coherence-based protection scheme”. Section “Experimental model” introduces a practical model for investigating the protection method. In Section “Test results analysis”, the test findings are analyzed and interpreted. In Section “Algorithm effectiveness”, the estimate of the protection attributes, protection algorithm features, major contributions, and a comparison between the currently developed technique and other recently published techniques are provided. In Section “Algorithm effectiveness”, algorithm performance estimation, its advantages, and a comparison between the currently developed technique and other recently published techniques will be provided. Algorithm limitations are presented in Section “Algorithm limitations”, and major contributions are summarized in Section “Main contributions”. Finally, the main practical outcomes are summarized in Section “Conclusions”.

## Coherence-based protection scheme

A series or shunt fault can cause a change in one of the power quality parameters, such as frequency, magnitude, phase shift, symmetry, or the waveform shape. In digital signals processing, the coherence coefficient is considered a proper estimator to measure the association degree and identify sudden changes between two waveforms/data sets^29,30^. These roles can be performed using the same estimation process in the same time. Besides, it allows for easy adjustments to the relay settings. Therefore, it is beneficial to establish an effective algorithm for windings protection of the AC machine using coherence criterion^31^. Many AC machines can be protected with this tool, like synchronous motors and generators, and induction motors and generators.

The method uses two kinds of coherence algorithms: (1) the cross-coherence algorithm, and (2) the auto-coherence algorithm^32–34^. The cross-coherence algorithm performs the functional role of digital differential current protection to classify and find out the internal shunt faults; while, the auto-coherence algorithm serves as an overcurrent protection to detect external, internal, and inter-turn faults. If one of the two algorithms fails, the other will respond to the fault conditions. Each algorithm is therefore redundant for the other, which improves the reliability of the protection scheme. The proposed methodology necessitates the construction of three single-phase current transformers situated at both neutral and load terminals of the AC machine windings. The numerical method based on the coherence criterion can diagnose faults on the AC machine stator windings, including but not limited to turn-to-turn, winding-to-neutral and winding-to-winding faults.

### Coherence estimators

#### Cross-coherence estimator

The cross-coherence estimator is a numerical method that can be used to estimate the relationship, as a function of frequency, between two different signals. The cross-coherence value ranges from 0.0 to + 1.0^29–31^. In this work, it is estimated between the neutral and power supply currents of each AC machine winding. The estimation is continually accomplished between each two corresponding data sets of the two different signals^32,33^. The selected data set is a single cycle of the fundamental power frequency of the system. It is possible to obtain three cross-coherence coefficients, namely *Ci*_*12a*_*, Ci*_*12b*_*,* and *Ci*_*12c*_, for the three-phase AC machine windings^30,34^. The arithmetic expression for the cross-coherence coefficient (*Ci*_*12x*_(*k*)) can be formulated between the neutral and supply currents (*i*_*1x*_(*n*) and* i*_*2x*_(*n*)) for each phase ‘*X*’ as follows^33^:

1 $$Ci_{12x} (k) = \frac{{\left[ {\left( {\sum\limits_{n = 0}^{N - 1} {I_{1x1} (k) \times I_{2x1} (k)} + I_{1x2} (k) \times I_{2x2} (k)} \right)^{2} + \left( {\sum\limits_{n = 0}^{N - 1} {I_{1x1} } (k) \times I_{2x2} (k) - I_{1x2} (k) \times I_{2x1} (k)} \right)^{2} } \right]}}{{\sum\limits_{n = 0}^{N - 1} {\left[ {(I_{1x1} (k))^{2} + (I_{1x2} (k))^{2} } \right]} \times \sum\limits_{n = 0}^{N - 1} {\left[ {(I_{2x1} (k))^{2} + (I_{2x2} (k))^{2} } \right]} }}$$

where

$$I_{1x1} (k) = \sum\limits_{n = 0}^{N - 1} {\left[ {i_{1x} (n) \cdot \cos \left( {\frac{2\pi kn}{N}} \right)} \right]}$$

$$I_{1x2} (k) = \sum\limits_{n = 0}^{N - 1} {\left[ {i_{1x} (n) \cdot \sin \left( {\frac{2\pi kn}{N}} \right)} \right]}$$

$$I_{2x1} (k) = \sum\limits_{n = 0}^{N - 1} {\left[ {i_{2x} (n) \cdot \cos \left( {\frac{2\pi kn}{N}} \right)} \right]}$$

$$I_{2x2} (k) = \sum\limits_{n = 0}^{N - 1} {\left[ {i_{2x} (n) \cdot \sin \left( {\frac{2\pi kn}{N}} \right)} \right]}$$

*I*_*1x1*_(*k*) is the cosine expression of the DFT for the wave *i*_*1x*_(*n*)*, I*_*1x2*_(*k*) is the sine expression of the DFT for the wave *i*_*1x*_(*n*)*, I*_*2x1*_(*k*) is the cosine expression of the DFT for the wave *i*_*2x*_(*n*)*, I*_*2x2*_(*k*) is the sine expression of the DFT for the wave *i*_*2x*_(*n*)*.*

#### Auto-coherence estimator

The auto-coherence estimator is a computational technique that can be employed to determine the relationship, as a function of frequency, between each pair of consecutive data sets of the same signal. Also, the auto-coherence value lies between 0.0 and + 1.0^35^. In this study, it can be calculated for each current signal taken at the neutral and power supply ends of the AC machine winding. The estimation is done between each two successive data sets, where the shifting time between them is one cycle of the fundamental power frequency^33–35^. In this work, the prescribed data set is one cycle, although, it is possible to select it as a sub-cycle. This pertains to the protection requirements and the prevailing conditions of the power system. The three-phase AC machine windings can have six auto-coherence coefficients (*Ci*_*1a*_*, Ci*_*1b*_*, Ci*_*1c*_*, Ci*_*2a*_*, Ci*_*2b*_*,* and *Ci*_*2c*_) can be obtained^33–35^.

The mathematical expression for the auto-coherence coefficient (*Ci*_*1x*_(*k*)) can be defined for the supply current (*i*_*1x*_(*n*)) for each phase ‘*X*’ as follows^35^:

2 $$Ci_{1x} (k) = \frac{{\left[ {\left( {\sum\limits_{n = 0}^{N - 1} {I_{1x1} (k) \times I_{3x1} (k)} + I_{1x2} (k) \times I_{3x2} (k)} \right)^{2} + \left( {\sum\limits_{n = 0}^{N - 1} {I_{1x1} } (k) \times I_{3x2} (k) - I_{1x2} (k) \times I_{3x1} (k)} \right)^{2} } \right]}}{{\sum\limits_{n = 0}^{N - 1} {\left[ {(I_{1x1} (k))^{2} + (I_{1x2} (k))^{2} } \right]} \times \sum\limits_{n = 0}^{N - 1} {\left[ {(I_{3x1} (k))^{2} + (I_{3x2} (k))^{2} } \right]} }}$$

$$I_{3x1} (k) = \sum\limits_{n = 0}^{N - 1} {\left[ {i_{1x} (n - N_{c} ).\cos \left( {\frac{{2\pi k(n - N_{c} )}}{N}} \right)} \right]}$$

$$I_{3x2} (k) = \sum\limits_{n = 0}^{N - 1} {\left[ {i_{1x} (n - N_{c} ).\sin \left( {\frac{{2\pi k(n - N_{c} )}}{N}} \right)} \right]}$$

*I*_*3x1*_(*k*) is the cosine expression of the DFT for the wave *i*_*1x*_(*n-N*_*c*_)*, I*_*3x2*_(*k*) is the sine expression of the DFT for the wave *i*_*1x*_(*n-N*_*c*_)*.*

Also, the mathematical expression for the auto-coherence coefficient (*Ci*_*2x*_(*k*)) can be obtained for the neutral current (*i*_*2x*_(*n*)) for each phase ‘*X*’ as follows^35^:

3 $$Ci_{2x} (k) = \frac{{\left[ {\left( {\sum\limits_{n = 0}^{N - 1} {I_{1x1} (k) \times I_{4x1} (k)} + I_{1x2} (k) \times I_{4x2} (k)} \right)^{2} + \left( {\sum\limits_{n = 0}^{N - 1} {I_{1x1} } (k) \times I_{4x2} (k) - I_{1x2} (k) \times I_{4x1} (k)} \right)^{2} } \right]}}{{\sum\limits_{n = 0}^{N - 1} {\left[ {(I_{1x1} (k))^{2} + (I_{1x2} (k))^{2} } \right]} \times \sum\limits_{n = 0}^{N - 1} {\left[ {(I_{4x1} (k))^{2} + (I_{4x2} (k))^{2} } \right]} }}$$

$$I_{4x1} (k) = \sum\limits_{n = 0}^{N - 1} {\left[ {i_{2x} (n - N_{c} ).\cos (\frac{{2\pi k(n - N_{c} )}}{N})} \right]}$$

$$I_{4x2} (k) = \sum\limits_{n = 0}^{N - 1} {\left[ {i_{2x} (n - N_{c} ).\sin \left( {\frac{{2\pi k(n - N_{c} )}}{N}} \right)} \right]}$$

*I*_*4x1*_(*k*) is the cosine expression of the DFT for the wave *i*_*2x*_(*n-N*_*c*_)*, I*_*4x2*_(*k*) is the sine expression of the DFT for the wave *i*_*2x*_(*n-N*_*c*_)*.*

The subscript* X* is the phase designation *A, B,* or *C*.

### Coherence-based faults detection and discrimination

Table 1 lists the operation rules of the coherence algorithm and its actions. It contains the protection action according to the situation type.

**Table 1:** The operation rules of the coherence algorithm and its actions.

Sr. no.	Coherence coefficients domain for phase *X* winding	The phase *X* winding condition	
	The setting deviations (*Δs*_*1*_ and* Δs*_*2*_) of the coherence are 0.05		
**1**	*1.0* − *Δs*_*1*_ ≤ *Ci*_*12x*_ ≤ *1.0,* and *1.0* − *Δs*_*2*_ ≤ *Ci*_*1x*_ ≤ *1.0,* and *1.0* − *Δs*_*2*_ ≤ *Ci*_*2x*_ ≤ *1.0*	Healthy phase *X* (Normal operating condition or external shunt fault for the phase *X*)	
		Blocking action for the equipment circuit breaker(s)	
**2**	*Ci*_*12x*_ < *1.0* − *Δs*_*1*_*,* and *Ci*_*1x*_ < *1.0* − *Δs*_*2*_*,* or *Ci*_*2x*_ < *1.0* − *Δs*_*2*_	Internal shunt fault for the phase *X*	
		Tripping action for the equipment circuit breaker(s)	
**3**	*1.0* − *Δs*_*1*_ ≤ *Ci*_*12x*_ ≤ *1.0,* and *0.0* ≤ *Ci*_*1x*_ < *1.0* − *Δs*_*2*_*,* or *0.0* ≤ *Ci*_*2x*_ < *1.0* − *Δs*_*2*_	External shunt fault, or Inter-turn fault for the phase *X*	
		Blocking action for the equipment circuit breaker(s) in the case of external shunt fault for the phase *X*	Tripping action for the equipment circuit breaker(s) in the case of inter-turn fault for the phase *X*

### Coherence-based tripping characteristic curve

**Fig. 1:**
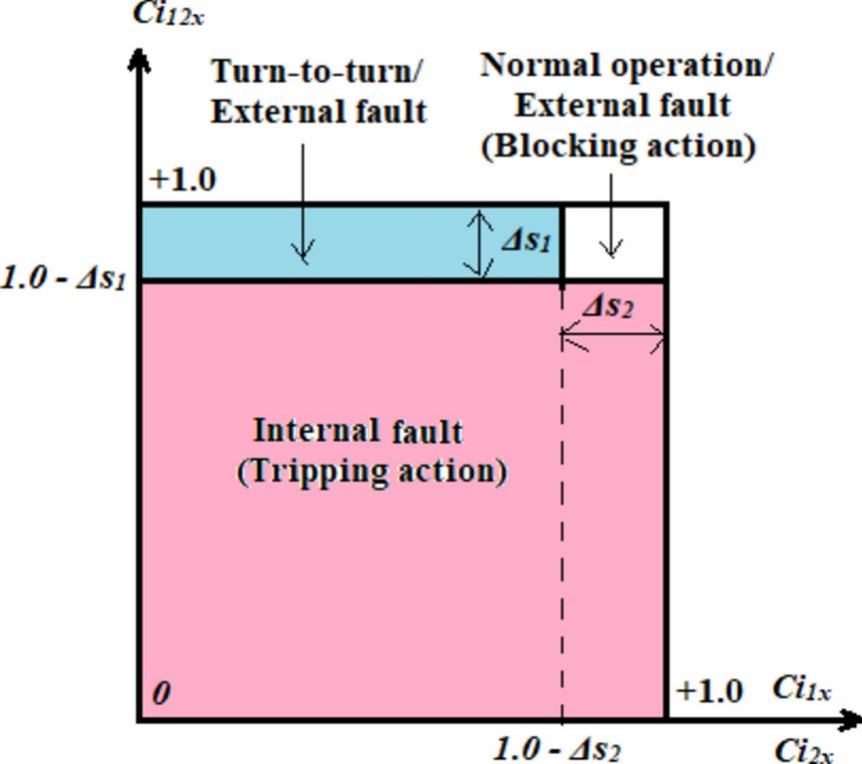
Tripping-characteristic curve based on the three coherence estimators (*Ci*_*12x*_*, Ci*_*1x*_*,* and *Ci*_*2x*_).

The numerical approach functions individually for each phase stator winding. The tripping-characteristic curve based on the coherence estimators (*Ci*_*12x*_*, Ci*_*1x*_*,* and *Ci*_*2x*_) is presented in Fig. 1. The curve is quadratic and can be divided into three regions that are designated as follows:

(I) Normal operating condition/External fault region: The blocking decision is taken to constrain isolating the AC motor circuit breaker(s),
(II) Turn-to-turn/External fault region: The tripping decision is dispatched to isolate the AC motor circuit breaker(s) in the event of turn-to-turn faults, while the blocking action is taken in the case of external faults, and
(III) Internal shunt fault region: The tripping decision is issued to isolate the AC motor circuit breaker(s) to protect the machine.

### Coherence-based inverse time curve

The author suggests a new mathematical expression for estimating the tripping time in the case of turn-to-turn faults. This expression necessitates the inclusion of the actual auto-coherence estimator (*Ci*_*1x*_ or *Ci*_*2x*_), the coherence pickup (*C*_*pu*_), and the time multiplier (*K*_*s*_). Equations (4, 5) can be used to estimate the operating times (*T*_*1op*_ and *T*_*2op*_), respectively.

4 $$T_{1op} = \frac{Ks}{{\left| {\left( {\frac{{C_{pu} }}{{Ci_{1x} }}} \right) - 1} \right|}}$$

5 $$T_{2op} = \frac{Ks}{{\left| {\left( {\frac{{C_{pu} }}{{Ci_{2x} }}} \right) - 1} \right|}}$$

where *T*_*1op*_: The operating time (in Sec) estimated using the auto-coherence (*Ci*_*1x*_), *T*_*2op*_: The operating time (in Sec) estimated using the auto-coherence (*Ci*_*2x*_), *K*_*s*_: The selected time multiplier, *C*_*pu*_: The coherence pickup of the algorithm (it is *C*_*pu*_ = *1.0* − *Δs*_*2*_ = *0.95*), *Ci*_*1x:*_ The auto-coherence estimator computed for the phase current *i*_*1x*_, and *Ci*_*2x:*_ The auto-coherence estimator quantified for the phase current *i*_*2x*_.

**Fig. 2:**
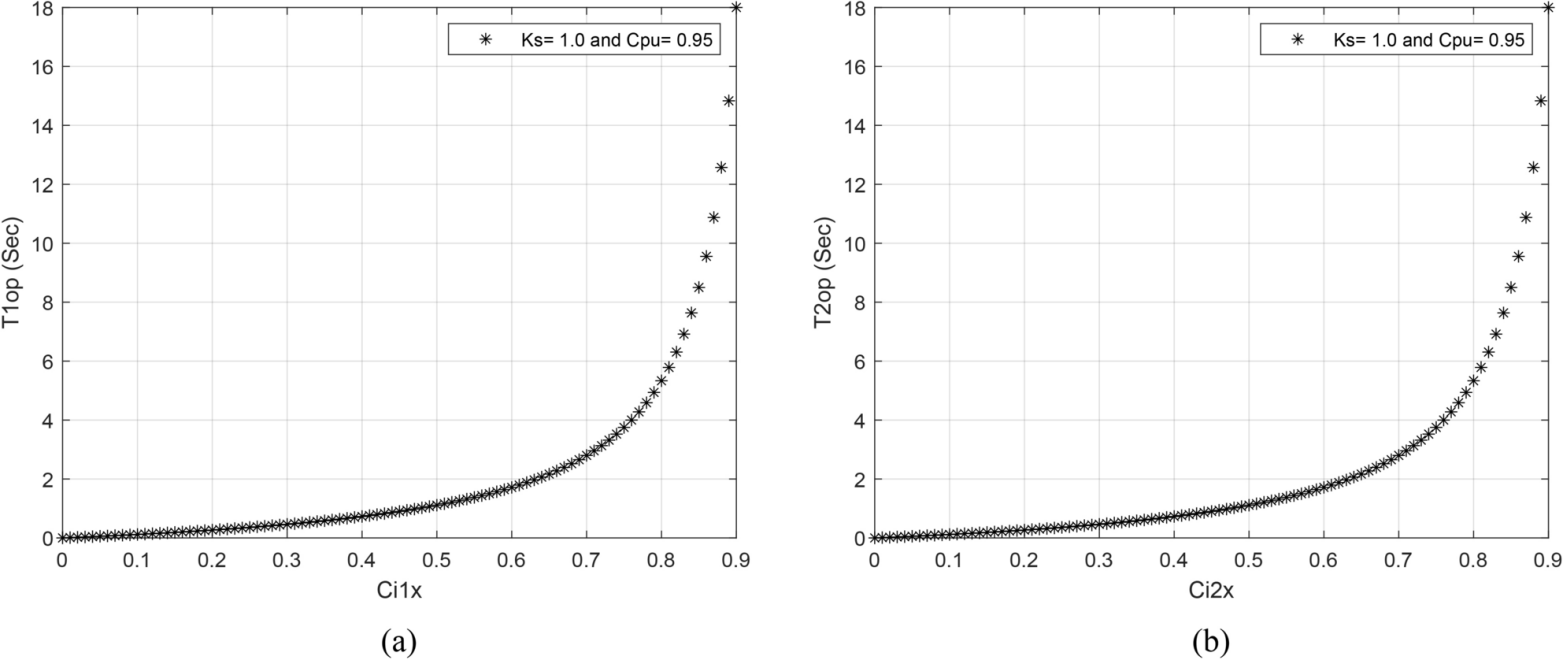
(**a**,**b**) The suggested auto-coherence-based tripping time curves for estimating the inter-turn fault tripping times (*T*_*1op*_ and *T*_*2op*_) when* K*_*s*_ = 1.0 and *C*_*pu*_ = 0.95. (**a**) *Ci*_*1x-T1op curve*_, and (**b**) *Ci*_*2x*_*-T2op curve*.

Figures 2a,b present the suggested auto-coherence-inverse time curves for estimating the inter-turn fault tripping times (*T*_*1op*_ and *T*_*2op*_) when* K*_*s*_ = 1.0 and *C*_*pu*_ = 0.95. The setting values of *K*_*s*_ and *C*_*pu*_ are based on the prevailing conditions of the system.

Table 2 illustrates the tripping time of the algorithm using the coherence criterion. As given in the table, the tripping time is infinite in the case of normal operations, it is instantaneous in the case of internal faults, and its tripping time curve takes the inverse characteristic in the case of turn-to-turn faults. The pragmatic operating time of the protection should originate after the motor starting time to assure the turn-to-turn fault event.

**Table 2:** The tripping time estimation based on the coherence criterion.

Machine condition	Coherence coefficients domain for phase *X* winding		Tripping time (in Seconds)
Normal operation/external fault	*1.0* − *Δs*_*1*_ ≤ *Ci*_*12x*_ ≤ *1.0,* and *1.0* − *Δs*_*2*_ ≤ *Ci*_*1x*_ ≤ *1.0,* and *1.0* − *Δs*_*2*_ ≤ *Ci*_*2x*_ ≤ *1.0*		**∞**
Internal shunt fault	*Ci*_*12x*_ < *1.0* − *Δs*_*1*_*,* and *Ci*_*1x*_ < *1.0* − *Δs*_*2*_*,* or *Ci*_*2x*_ < *1.0* − *Δs*_*2*_		Instantaneous
Turn-to-turn fault	*1.0* − *Δs*_*1*_ ≤ *Ci*_*12x*_ ≤ *1.0,* and *0.0* ≤ *Ci*_*1x*_ < *1.0* − *Δs*_*2*_*,* or 0.0 ≤ Ci_2x_ < 1.0 − Δs_2_	When* Ci*_*1x*_ < (*1.0* − *Δs*_*2*_) $T_{1op} = \frac{{K_{s} }}{{\left| {\frac{{1.0 - \Delta x_{2} }}{{Ci_{1x} }}^{{}} - 1} \right|}}$ (6) When* Ci*_*2x*_ < (*1.0* − *Δs*_*2*_) $T_{2op} = \frac{{K_{s} }}{{\left| {\frac{{1.0 - \Delta x_{2} }}{{Ci_{2x} }} - 1} \right|}}$ (7)	When* Ci*_*1x*_ ≥ (*1.0* − *Δs*_*2*_) $T_{1op} = \frac{{K_{s} }}{{\left| {\frac{{1.0 - \Delta x_{2} }}{{Ci_{1x} }} - 1} \right|}} = \propto$ (8) When* Ci*_*2x*_ ≥ (*1.0* − *Δs*_*2*_) $T_{2op} = \frac{{K_{s} }}{{\left| {\frac{{1.0 - \Delta x_{2} }}{{Ci_{2x} }} - 1} \right|}} = \propto$ (9)

### Coherence-based protection strategy

1. Take the measurements of the supply and neutral currents (*i*_*1x*_ and *i*_*2x*_) at both terminals of each AC motor stator winding,
2. Transform the analog current waves into discrete values using a digital DAC,
3. Specify the sampling rate per cycle (*N*_*c*_) of the fundamental power frequency, as well as the sampling rate per the data set (*N*_*s*_),
4. Select the coherence setting deviations (*Δs*_*1*_*,* and *Δs*_*2*_). Where,
  *Δs*_*1*_ is the specified setting of the cross-coherence coefficient (*Ci*_*12x*_), which is used to detect internal shunt faults, and.
  *Δs*_*2*_ is the specified setting of the auto-coherence coefficient (*Ci*_*1x*_ or* Ci*_*2x*_), which is used to define all kinds of fault, including turn-to-turn, external, and internal faults.
5. Compute the coherence coefficients (*Ci*_*12x*_*, Ci*_*1x*_*,* and *Ci*_*2x*_*,*) for the current signals taken from each phase ‘*X*’,
6. Apply the operation rules of the coherence algorithm for each phase (as indicated in Table 1),
7. Execute the protection algorithm action as follows:
  - Blocking action for the equipment circuit breaker(s) in the cases of healthy phase *X*.
  - Blocking action for the equipment circuit breaker(s) in the cases of external fault for the phase *X*.
  - Tripping action for the equipment circuit breaker(s) in the cases of internal shunt fault for the phase *X.*
  - Tripping action for the equipment circuit breaker(s) in the cases of turn-to-turn fault for the phase *X*.
8. Initiate a tripping action to the machine circuit breaker(s) when the machine is experiencing turn-to-turn or internal shunt faults.

**Fig. 3:**
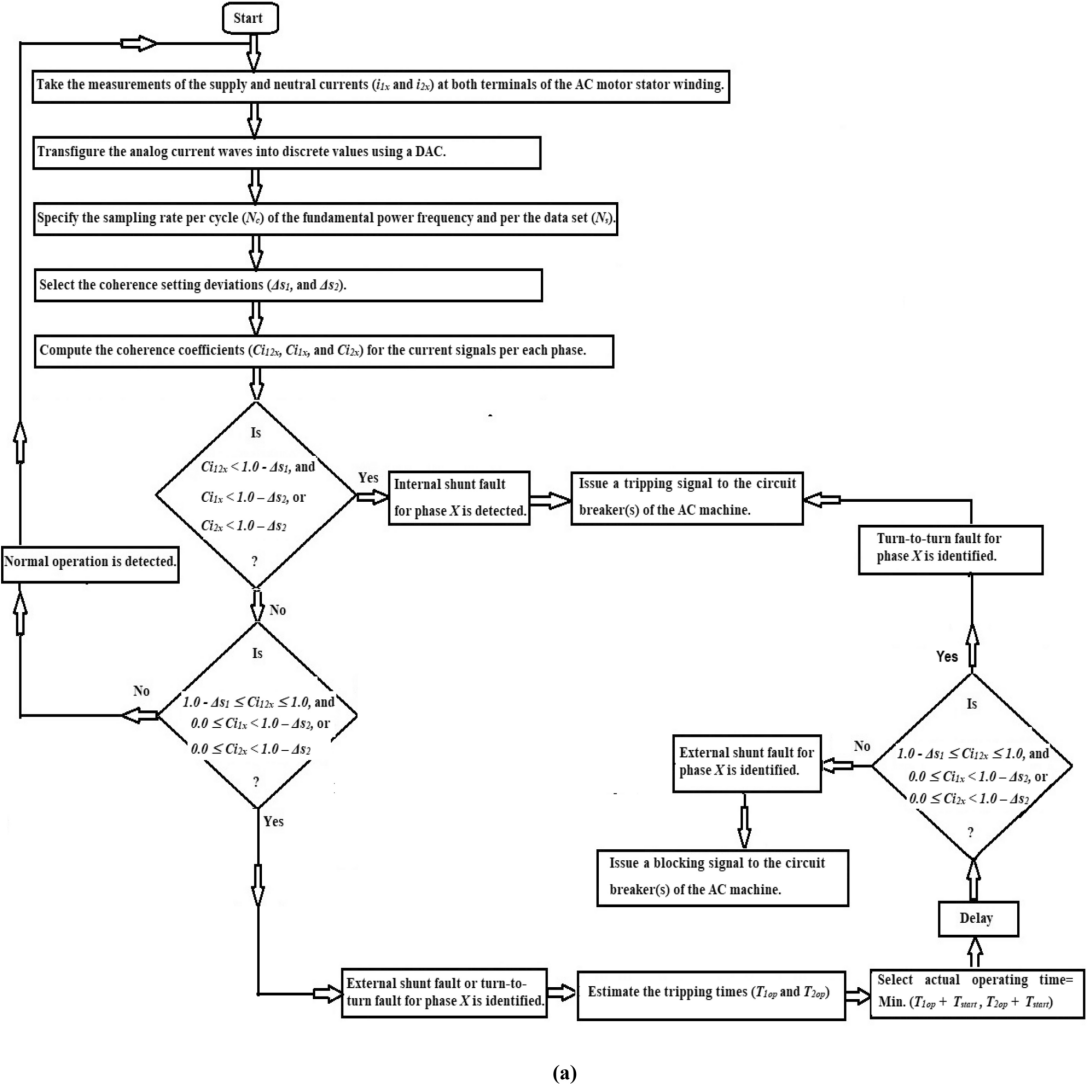

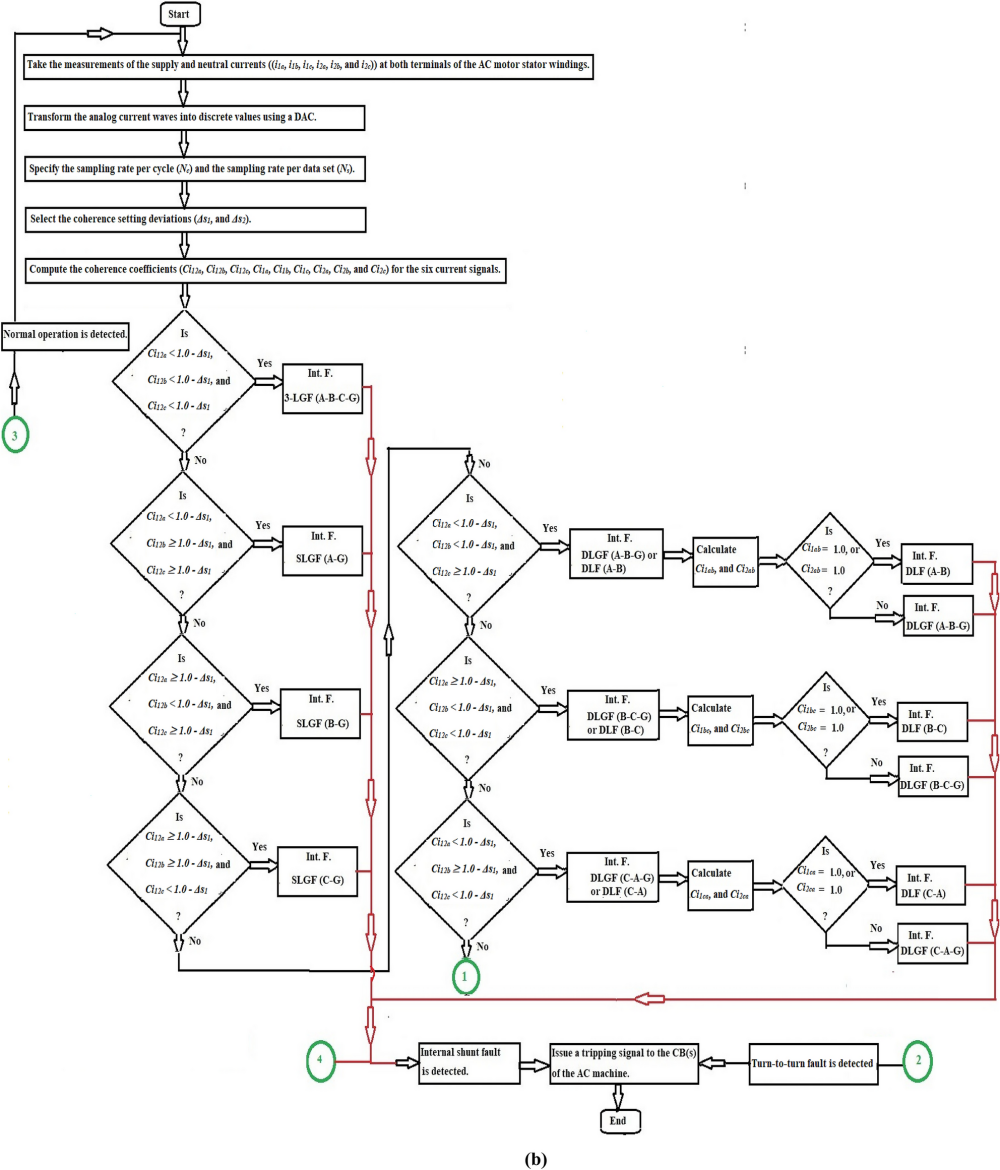

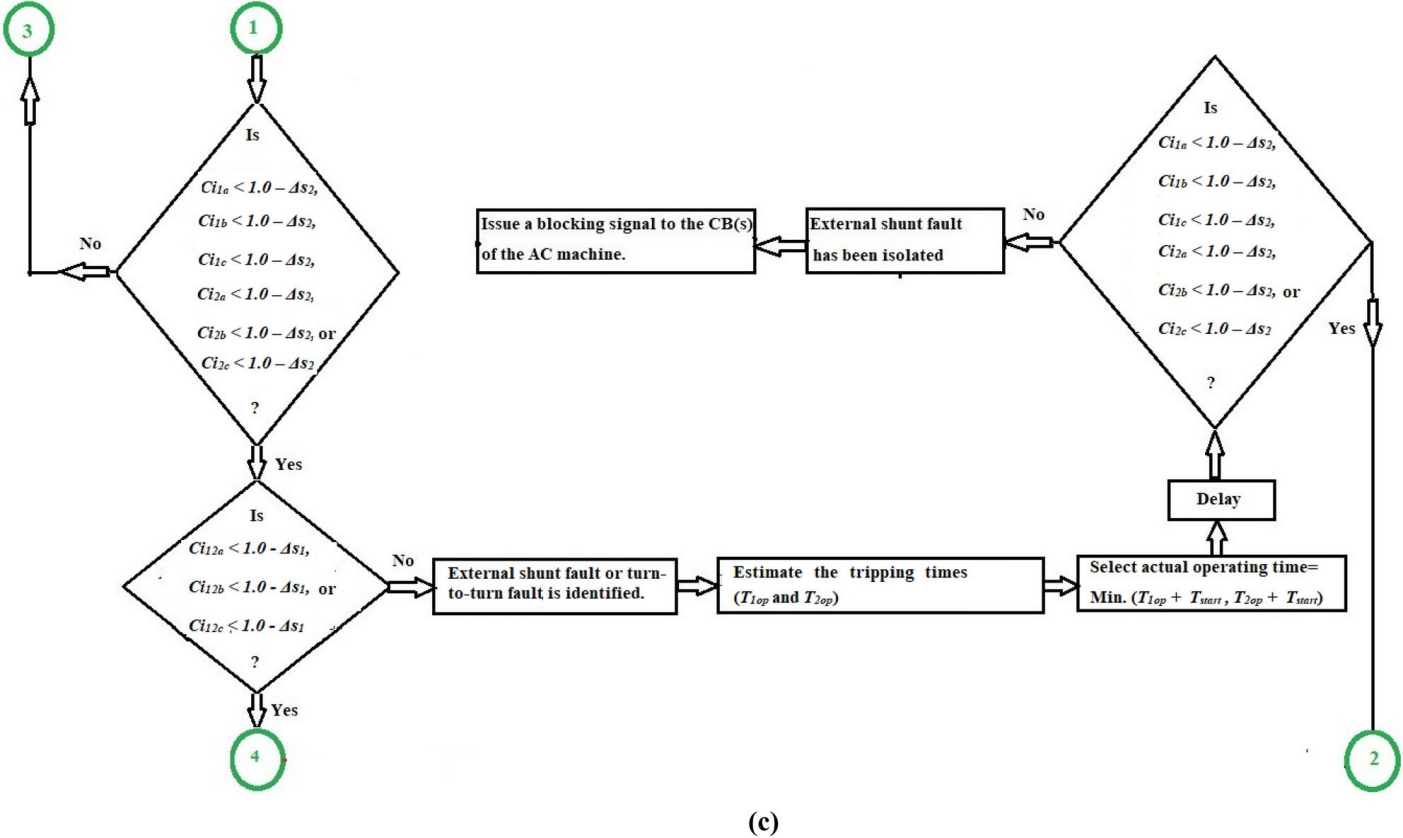
(**a**) Flow chart of the fault detection using the three coherence coefficients for each phase stator winding of the AC machine. (**b**) Flow chart of the fault classification and discrimination using the coherence coefficients for the three phase stator windings of the AC machine. (**c**) Flow chart of the fault classification and discrimination using the coherence coefficients for the three phase stator windings of the AC machine (Continued).

The flow chart of the protection mechanism for detecting and differentiating between inter-turn, external and internal faults (for each phase) is developed in Fig. 3a. The protection strategy can be processed in the manner described below.

In this study, the following factors can be taken into account:

- The algorithm can classify the four classifications of internal shunt faults (*3LGF, DLF, DLGF,* and *SLGF*)*.* As shown in Fig. 3b,c, the internal fault can be determined by monitoring sudden drops that happen in the three phase cross-coherence coefficients (*Ci*_*12a*_*, Ci*_*12b*_ and *Ci*_*12c*_).
- The algorithm can discriminate between ground faults (*DLGF* and *SLGF*) and phase faults (*3LGF* and *DLF*), as illustrated in Fig. 3b,c.
- The algorithm can differentiate between unsymmetrical faults (*DLF, DLGF,* and *SLGF*) and symmetrical faults (*3LGF*), as depicted in Fig. 3b,c.
- The algorithm can distinguish between the turn-to-turn faults located on the AC motor windings and the external faults situated in the adjacent protection zone. For external faults, the primary protection of the next zone separates them. Otherwise, the fault category will be eventually turn-to-turn on the motor windings, which should be avoided by disconnecting the system, and
- The algorithm can discriminate between the faulty current and the starting current of the AC motor.

### Computational time adjustment

As mentioned before, the coherence estimators apply the data window concept, which has effects on the computational time and the fault detection time. Consequently, the technique speed can be easily controlled by selecting the data window size that is lower than or equal to one cycle of the fundamental power frequency. The data set can be selected as being one, 3/4, 1/2, or 1/4 of the cycle. This is related to the requirements of the protection and the prevailing conditions of the system.

The response time of the microprocessor relay is directly related to the following:

1. Its microprocessor (MP) speed,
2. The number of lines per computer code (i.e., the amount of programming), and
3. The additional features or complexity level of computer programming.

### Coherence settings selection

In this study, the setting values of the coherence estimators have been verified on a practical model of an induction machine with tapped windings. The settings have been practically adjusted and investigated by watching the alarm flag and relay response signal under normal operating conditions in the experimental setup. This has been accomplished with the aid of LABVIEW software. The threshold values in the LABVIEW program have been modified to pass the acceptable unbalance and normal operating conditions. These settings are easily adjustable with any new experimental setup, depending on how much unbalance of three-phase waveforms is required. This is a significant advantage of the proposed algorithm to increase the reliability of the system.

Furthermore, the actual values of the coherence coefficients are compared to the coherence settings. Practically, the effects of measurement errors, instrument transformer linear errors, DC components, decent harmonics, and transient faults can be avoided using reasonable data window and coherence settings. These settings have the ability to tune the protection requirements, such as security, dependability, sensitivity and speed. Therefore, the protection decision is contingent upon the running coherence quantities and the threshold values of the system. The output decision can be sent to trip the machine circuit breaker(s) and to establish an alarm flag.

### Avoiding the motor starting current

It is well known that the motor starting current (*I*_*startup*_) may exceed 8.0 times of its nominal current. To differentiate between the overcurrent and starting current of the motor, it is recommended to incorporate a time element to prevent the protection operation when the AC motor starts. The time element will trip machine circuit breaker(s) when the overcurrent event occurs beyond the machine starting time (*T*_*startup*_). In other words, the tripping time should be initiated after the motor starting time. To avoid incorrect operation of the algorithm, the starting current–time curve of the AC motor should be provided in the protection algorithm.

### Algorithm sensitivity

The sensitivity of the present protection depends on the running coherence estimator (*Ci*_*1x*_ or *Ci*_*2x*_) and the coherence pickup value (*C*_*pu*_), as given in Eqs. (10, 11).

10 $$Sensitivity\;\alpha \;\frac{{C_{pu} }}{{Ci_{1x} }} = \frac{{(1.0 - \Delta x_{2} )}}{{Ci_{1x} }}$$

or

11 $$Sensitivity\;\alpha \;\frac{{C_{pu} }}{{Ci_{2x} }} = \frac{{(1.0 - \Delta x_{2} )}}{{Ci_{2x} }}$$

## Experimental model

**Fig. 4:**
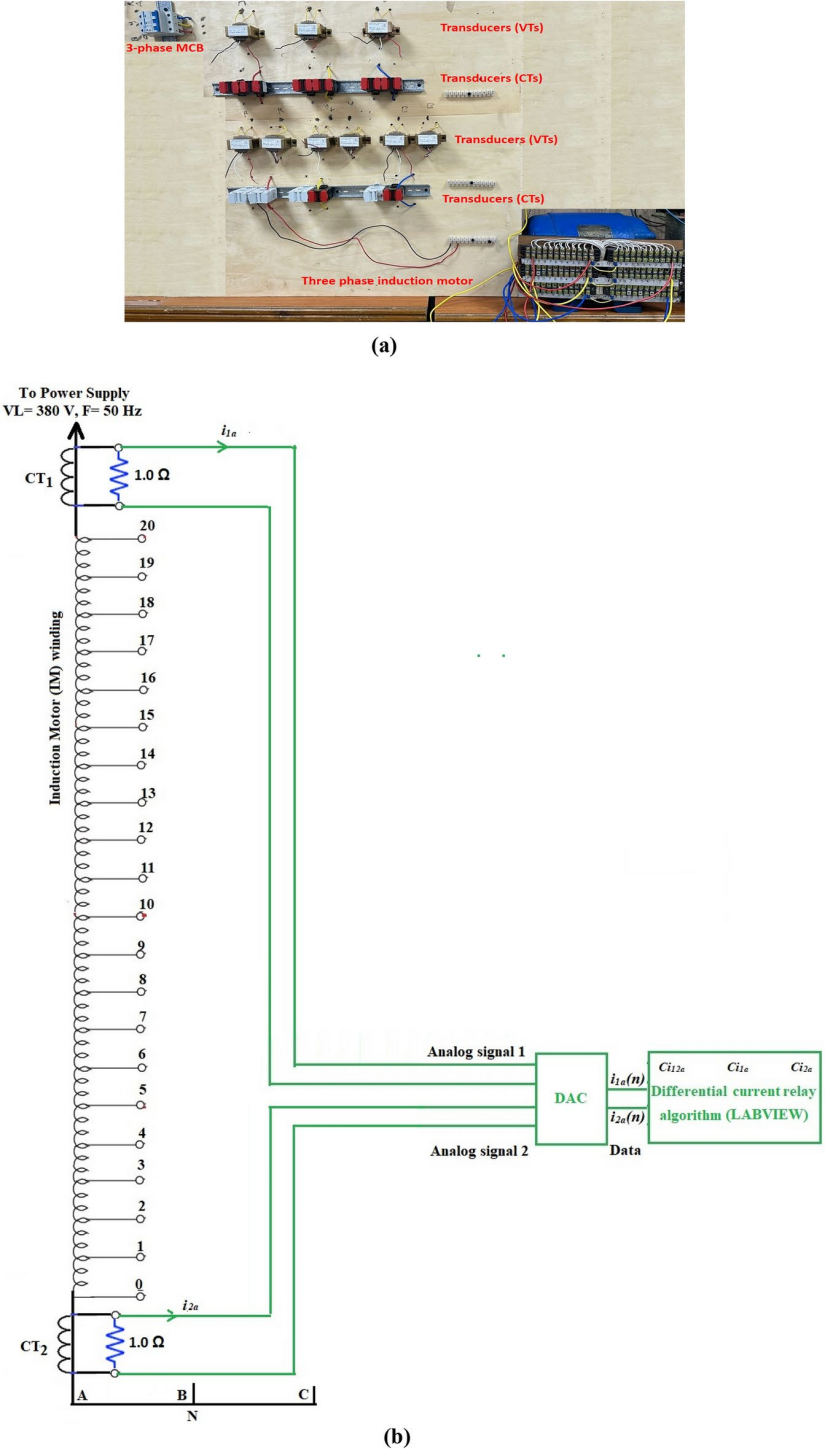
(**a**) Experimental power model. (**b**) The connection circuit diagram of the AC machine with 20 taps per each phase stator winding.

The experimental model is composed of a three-phase power source with a voltage rating of *V*_*L*_ = 380.0 V, a three-phase circuit breaker with a current rating of 63.0 A, a three-phase induction machine with a power rating of 2.9 kW, and two current transformers placed at the supply and neutral terminals of each winding of the machine. Each current transformer has a turns’ ratio of 200/5, and its burden is 1 Ω. The three-phase stator windings of the induction motor have been re-winded, where each winding has 20 taps per phase. The taps arrangement of the machine windings aims to conduct comprehensive pragmatic tests under different types of faults to examine the methodology’s effectiveness. It is therefore possible to implement a variety of series and shunt faults on the windings, such as turn-to-turn, phase-to-phase, and phase-to-ground faults. Also, this arrangement facilitates building transducers at the neutral and supply sides for each stator winding of the machine. A photo of the three-phase AC motor with 20 taps per each phase winding is shown in Fig. 4a. The connection circuit diagram of the AC machine with 20 taps per each stator winding, two current transformers per each phase, and an analog-to-digital converter, and a personal computer provided with LABVIEW software is depicted in Fig. 4b. Appendix 1 encompasses the specifications of the power model components.

## Test results analysis

In this work, Data Acquisition Card (DAC) and LABVIEW software are used to examine the protection approach under extensive types of inter-turn, external and internal faults. The DAC is manufactured by National Instruments USB-6009, which converts analog signals to digital measurements. It is set to operate in differential mode, with a sampling rate of 50.0 samples per cycle. Two current transformers for each phase are beneficial for measurement and protection purposes for the machine, which are constructed at both the power supply and neutral sides of each stator winding. The current signals obtained from the two current transformers are processed for estimating the three coherence coefficients (*Ci*_*12x*_*, Ci*_*1x*_*,* and *Ci*_*2x*_) for each phase ‘*X*’. Input quantities of the coherence-based protection algorithm are involved in Appendix 2.

Various testing examinations are conducted in different situations. The test results presented in this section are only for phase winding ‘*A*’ of the machine. Table 3 includes the testing results for thirty different types of faults. The table also illustrates protection actions, action types, and tripping times for the thirty tests.

**Table 3:** Different actual faults, algorithm actions (blocking/tripping), action type (correct/incorrect), and tripping time.

Case no	System fault type	Algorithm action (Blocking/tripping)	Action type (correct/incorrect)	Estimated tripping time (in milliseconds)
Case 1	Normal operation (Phase A)	Blocking	Correct	∞
Case 2	Motor starting (Phase A)	Blocking	Correct	∞
Case 3	TTTF (A1–A2) Phase A—Series Fault	Tripping	Correct	284
Case 4	TTTF (A1–A2) Phase A—Series Fault	Blocking	Incorrect	∞
Case 5	TTTF (A1–A3) Phase A—Series Fault	Tripping	Correct	234
Case 6	TTTF (A1–A4) Phase A—Series Fault	Tripping	Correct	158
Case 7	TTTF (A1–A5) Phase A—Series Fault	Tripping	Correct	145
Case 8	TTTF (A1–A7) Phase A—Series Fault	Tripping	Correct	0
Case 9	TTTF (A1–A10) Phase A—Series Fault	Tripping	Correct	118
Case 10	TTTF (B1–B10)—External Fault	Blocking	Correct	∞
Case 11	TTTF (C1–C10)—External Fault	Blocking	Correct	∞
Case 12	TTTF (B3–C3)—External Shunt Fault	Blocking	Correct	∞
Case 13	TTTF (B2–C2)—External Shunt Fault	Blocking	Correct	∞
Case 14	TTTF (B10–C10)—External Shunt Fault	Blocking	Correct	∞
Case 15	Internal Shunt Fault (A2–B2)	Tripping	Correct	instantaneous
Case 16	Internal Shunt Fault (A3–B3)	Tripping	Correct	instantaneous
Case 17	Internal Shunt Fault (A3–B3)	Tripping	Correct	instantaneous
Case 18	Internal Shunt Fault (A5–B5)	Tripping	Correct	instantaneous
Case 19	Internal Shunt Fault (A10–B10)	Tripping	Correct	instantaneous
Case 20	Internal Shunt Fault (A10–B10)	Tripping	Correct	instantaneous
Case 21	Internal Shunt Fault (A3–C3)	Tripping	Correct	instantaneous
Case 22	Internal Shunt Fault (A3–C3)	Tripping	Correct	instantaneous
Case 23	Internal Shunt Fault (A5–C5)	Tripping	Correct	instantaneous
Case 24	Internal Shunt Fault (A6–C6)	Tripping	Correct	instantaneous
Case 25	Internal Shunt Fault (A10–C10)	Tripping	Correct	instantaneous
Case 26	Internal Shunt Fault (A6–B6)	Tripping	Correct	instantaneous
Case 27	Internal Shunt Fault (A4–B4)	Tripping	Correct	instantaneous
Case 28	Internal Shunt Fault (A4–B4)	Tripping	Correct	instantaneous
Case 29	Internal Shunt Fault (A10–B10)	Tripping	Correct	instantaneous
Case 30	Internal Shunt Fault (A4–B4)	Tripping	Correct	instantaneous

### Case 1: Normal operation (Phase A)

**Fig. 5:**
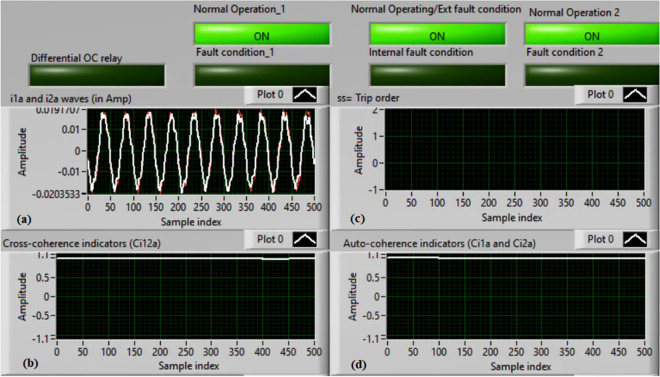
Results for case 1. (**a**) Two measured currents (i_1a_ and i_2a_), (**b**) Cross-coherence estimator (Ci_12a_), (**c**) Blocking signal, and (**d**) Auto-coherence estimators(Ci_1a_ and Ci_2a_).

Figures 5a–d present the experimental results for case 1. This case is healthy phase *A*. The two measured currents (*i*_*1a*_ and *i*_*2a*_) are similar, as shown in Fig. 5a. As seen in Fig. 5b, the cross-coherence estimator (*Ci*_*12a*_) is nearly 1.0. As a result, no internal shunt fault happens. In addition, both auto-coherence estimators (*Ci*_*1a*_*,* and *Ci*_*2a*_) are identical and roughly 1.0 (i.e., their values are greater than the coherence setting of 0.95), as demonstrated in Fig. 5d. Therefore, no turn-to-turn fault occurs. In this situation, the recorded results (for the ‘*A*’ phase stator winding) indicate that the motor operates normally, which leads to a blocking signal declaration (as presented in Fig. 5c).

### Case 2: Motor starting (Phase A)

**Fig. 6:**
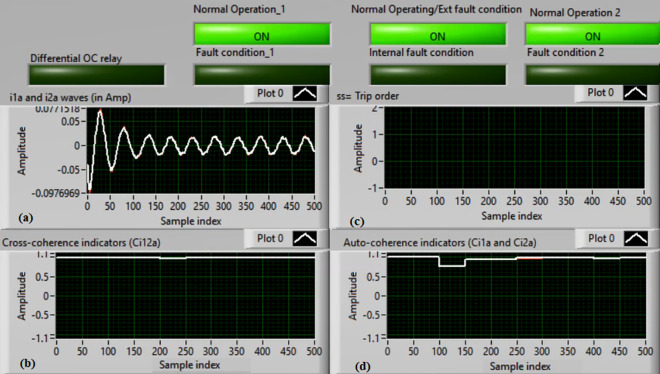
Results for case 2. (**a**) Two measured currents (i_1a_ and i_2a_), (**b**) Cross-coherence estimator (Ci_12a_), (**c**) Blocking signal, and (**d**) Auto-coherence estimators (Ci_1a_and Ci_2a_).

Figures 6a–d depict the experimental results for case 2. The results are recorded when a motor operation starts. The two secondary currents (*i*_*1a*_ and *i*_*2a*_) are corresponding, as demonstrated in Fig. 6a. As illustrated in Fig. 6b, the cross-coherence estimator (*Ci*_*12a*_) is almost 1.0. Therefore, no internal shunt fault exits for the ‘*A*’ phase stator winding. Whereas, both auto-coherence estimators (*Ci*_*1a*_*,* and *Ci*_*2a*_) are identical and lower than the coherence setting of 0.95, as shown in Fig. 6d. In the case of motor starting, the actual tripping time of the relay is beyond the motor start up time. The motor start up time is selected 5.0 cycles of the fundamental power frequency. As a result, no turn-to-turn fault is found for the ‘*A*’ phase stator winding. In this particular case, the obtained results (for the ‘*A*’ phase stator winding) confirm that the motor operation gets started, resulting in no tripping signal annunciation (as observed in Fig. 6c).

### Case 3: TTTF (A1–A2) Phase A

**Fig. 7:**
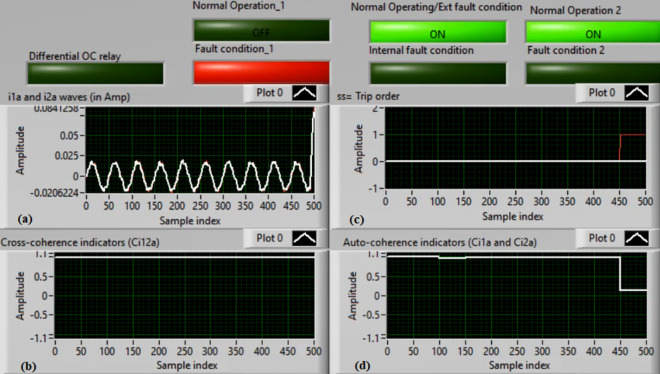
Results for case 3. (**a**) Two measured currents (i_1a_ and i_2a_), (**b**) Cross-coherence estimator (Ci1_2a_), (**c**) Blocking signal, and (**d**) Auto-coherence estimators (Ci_1a_and Ci_2a_).

Figures 7a–d show the experimental results for case 3. This case is turn-to-turn fault (*A1–A2*). As shown in Fig. 7a, the two currents (*i*_*1a*_ and *i*_*2a*_) are symmetrical. As depicted in Fig. 7b, the cross-coherence coefficient (*Ci*_*12a*_) is approximately 1.0. Thus, no internal shunt fault is observed. But both auto-coherence coefficients (*Ci*_*1a*_*,* and *Ci*_*2a*_) are symmetrical and less than the coherence setting of 0.95, as presented in Fig. 7d. As a consequence, a turn-to-turn fault is detected on the ‘*A*’ phase stator winding of the induction motor. In this instance, the algorithm results (for the ‘*A*’ phase stator winding) verify that the motor state is abnormal due to the inter-turn fault occurrence, causing a tripping signal declaration (as revealed in Fig. 7c).

### Case 4: TTTF (A1–A2) Phase A

**Fig. 8:**
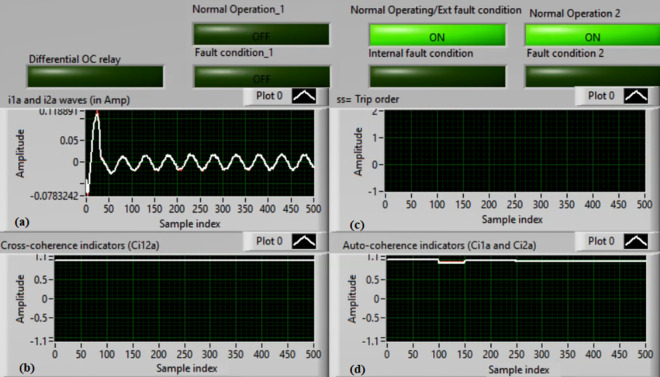
Results for case 4. (**a**) Two measured currents (i_1a_ and i_2a_), (**b**) Cross-coherence estimator (Ci1_2a_), (**c**) Blocking signal, and (**d**) Auto-coherence estimators (Ci_1a_and Ci_2a_).

Figures 8a–d present the experimental results for case 4. This case is turn-to-turn fault (*A1–A2*). In this event, the relay has failed to send a tripping flag. However, the relay has issued a correct action in the previous case study for the same TTTF (*A1–A2*). As demonstrated in Fig. 8a, the two currents (*i*_*1a*_ and *i*_*2a*_) are identical. As illustrated in Fig. 8b, the cross-coherence coefficient (*Ci*_*12a*_) is nearly 1.0. Consequently, no internal shunt fault arises. But both auto-coherence coefficients (*Ci*_*1a*_*,* and *Ci*_*2a*_) are identical and larger than the coherence pickup value of 0.95, as introduced in Fig. 8d. Because of this, the proposed algorithm fails to detect the turn-to-turn fault located on the ‘*A*’ phase stator winding of the induction machine. A blocking signal is declared in this test, as shown in Fig. 8c. It is considered an incorrect action for the proposed protection.

### Case 5: TTTF (A1–A3) Phase A

**Fig. 9:**
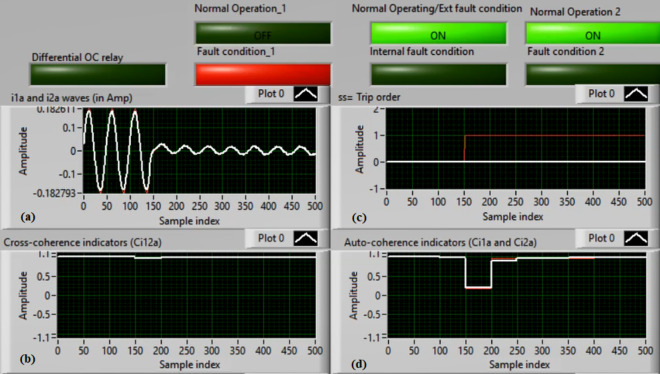
Results for case 5. (**a**) Two measured currents (i_1a_ and i_2a_), (**b**) Cross-coherence estimator (Ci1_2a_), and (**c**) Tripping signal, and (**d**) Auto-coherence estimators(Ci_1a_ and Ci_2a_).

Figures 9a–d illustrate the experimental results for case 5. This case is turn-to-turn fault (*A1–A3*). As described in Fig. 9a, the two currents (*i*_*1a*_ and *i*_*2a*_) are roughly equal. As presented in Fig. 9b, the cross-coherence coefficient (*Ci*_*12a*_) is about 1.0. Therefore, no internal shunt fault is situated on the ‘*A*’ phase machine stator winding. Whereas, both auto-coherence coefficients (*Ci*_*1a*_*,* and *Ci*_*2a*_) are commensurate and below the coherence setting of 0.95, as shown in Fig. 9d. Thus, a turn-to-turn fault is originated on the ‘*A*’ phase motor stator winding. In this case study, the generated results reveal that the motor current waves (for the ‘*A*’ phase stator winding) are faulty because of the turn-to-turn fault presence. A tripping signal is sent to the declaration board as results of this, as shown in Fig. 9c.

### Case 6: TTTF (A1–A4) Phase A

**Fig. 10:**
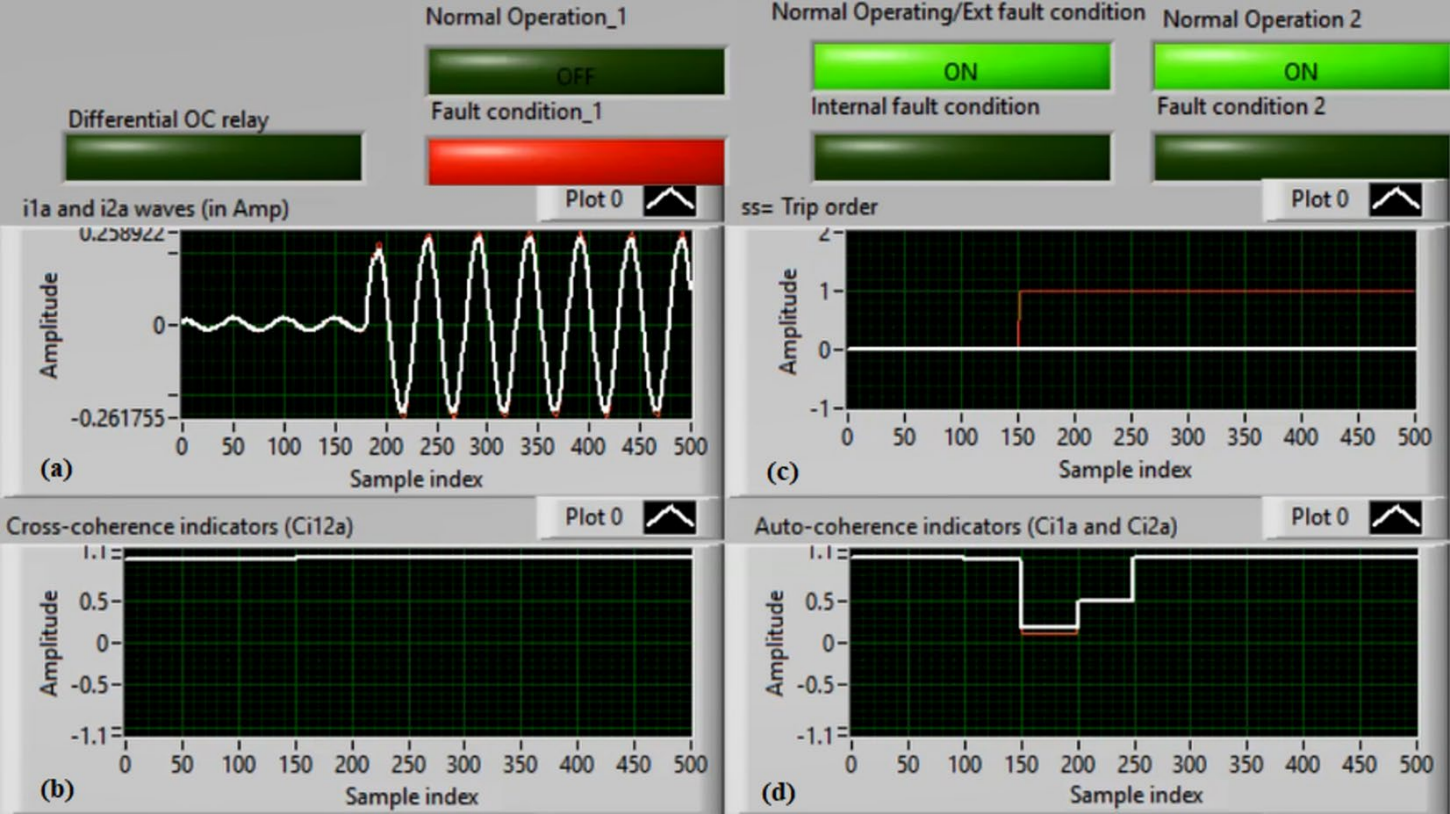
Results for case 6. (**a**) Two measured currents (i_1a_ and i_2a_), (**b**) Cross-coherence estimator (Ci1_2a_), (**c**) Tripping signal, and (**d**) Auto-coherence estimators(Ci_1a_ and Ci_2a_).

Figures 10a–d show the experimental results for case 6. This instance is turn-to-turn fault (*A1–A4*). The two measured currents (*i*_*1a*_ and *i*_*2a*_) are congruent, as demonstrated in Fig. 10a. As exhibited in Fig. 10b, the cross-coherence quantity (*Ci*_*12a*_) is close to 1.0. Consequently, no internal shunt fault is present. In contrast, both auto-coherence quantities (*Ci*_*1a*_*,* and *Ci*_*2a*_) are conforming and beneath the coherence threshold value of 0.95, as given in Fig. 10d. Accordingly, the inter-turn fault is figured out on the ‘*A*’ phase stator winding of this equipment. Under this circumstance, the testing results demonstrate that the machine current waveforms are raised remarkably because of the turn-to-turn fault incident. Subsequently, a tripping flag is issued to the alarm board, as illustrated in Fig. 10c.

### Case 7: TTTF (A1–A5) Phase A

**Fig. 11:**
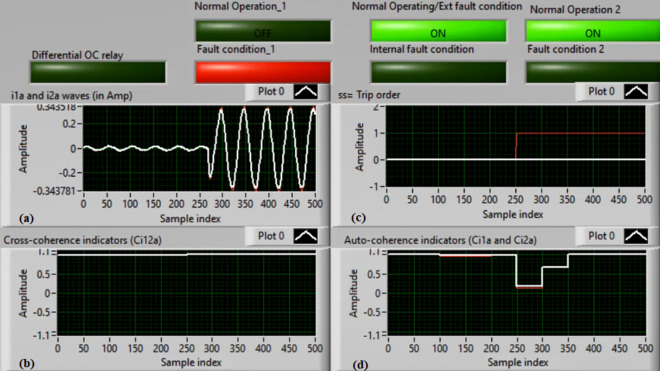
Results for case 7. (**a**) Two measured currents (i_1a_ and i_2a_), (**b**) Cross-coherence estimator (Ci1_2a_), and (**c**) Tripping signal, and (**d**) Auto-coherence estimators(Ci_1a_ and Ci_2a_).

Figure 11a–d depict the experimental results for case 7. This scenario is turn-to-turn fault (*A1–A5*). The current signals (*i*_*1a*_ and *i*_*2a*_) are correspondent, as illustrated in Fig. 11a. As shown in Fig. 11b, the cross-coherence measure (*Ci*_*12a*_) is around unity. As a consequence, there is no internal shunt fault. In contrast, the two auto-coherence estimators (*Ci*_*1a*_*,* and *Ci*_*2a*_) are congruous and lower than the prescribed setting of the coherence, as depicted in Fig. 11d. Subsequently, a TTTF is present on the ‘*A*’ phase stator winding of the AC motor. In this scenario, the algorithm outcomes illustrate that the motor current values rise significantly owing to the TTTF presence. Accordingly, a tripping signal is sent to the alarm panel, as exhibited in Fig. 11c.

### Case 8: TTTF (A1–A7) Phase A

**Fig. 12:**
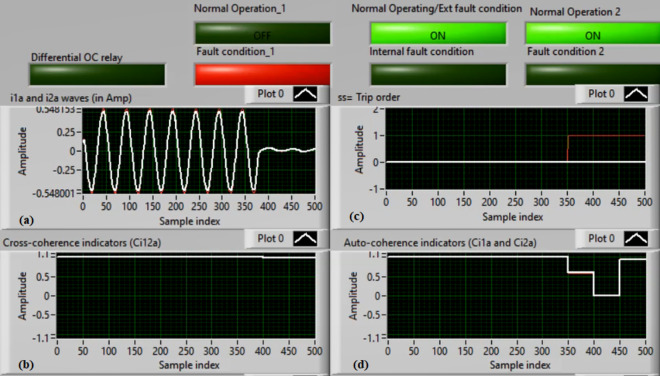
Results for case 8. (**a**) Two measured currents (i_1a_ and i_2a_), (**b**) Cross-coherence estimator (Ci1_2a_), and (**c**) Tripping signal, and (**d**) Auto-coherence estimators(Ci_1a_ and Ci_2a_).

Figure 12a–d depict the experimental results for case 8. This occasion is turn-to-turn fault (*A1–A7*). The current waves (*i*_*1a*_ and *i*_*2a*_) are identical, as shown in Fig. 12a. As illustrated in Fig. 12b, the cross-coherence estimator (*Ci*_*12a*_) is nearly unity. Subsequently, there is no internal shunt fault. Whereas, the two auto-coherence estimators (*Ci*_*1a*_*,* and *Ci*_*2a*_) are lower than the predetermined coherence setting, and their values are identical, as plotted in Fig. 12d. Therefore, a TTTF is identified on the ‘*A*’ phase stator winding of the induction motor. In this experiment, the testing results show that the motor currents increase excessively as a result of the TTTF occurrence. Consequently, a tripping signal is transmitted to the declaration board, as described in Fig. 12c.

### Case 9: TTTF (A1–A10) Phase A

**Fig. 13:**
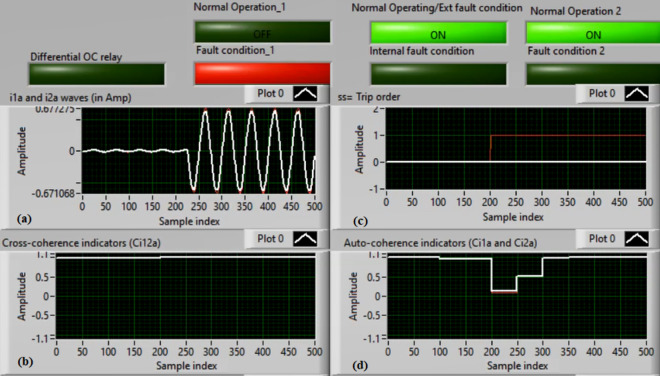
Results for case 9. (a) Two measured currents (i_1a_ and i_2a_), (**b**) Cross-coherence estimator (Ci1_2a_), and (**c**) Tripping signal, and (**d**) Auto-coherence estimators(Ci_1a_ and Ci_2a_).

Figure 13a–d exhibit the experimental results for case 9. This situation is turn-to-turn fault (*A1–A10*). The two currents (*i*_*1a*_ and *i*_*2a*_) are identical, as described in Fig. 13a. As depicted in Fig. 13b, the cross-coherence coefficient (*Ci*_*12a*_) is around unity. Therefore, there is no internal shunt fault. But the two auto-coherence coefficients (*Ci*_*1a*_*,* and *Ci*_*2a*_) are less than the coherence presetting, and their values are correspondent, as shown in Fig. 13d. Subsequently, a TTTF is already present on the ‘*A*’ phase stator winding of the motor. In this scenario, the recorded results reveal that the motor currents surge suddenly when the TTTF happens. Hence, a tripping signal is declared by the alarm panel, as illustrated in Fig. 13c.

#### Case 10: TTTF (B1–B10)

**Fig. 14:**
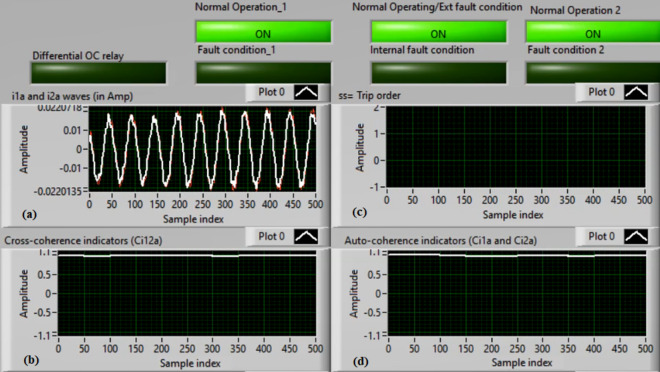
Results for case 10. (**a**) Two measured currents (i_1a_ and i_2a_), (**b**) Cross-coherence estimator (Ci1_2a_), and (**c**) Blocking signal, and (**d**) Auto-coherence estimators(Ci_1a_ and Ci_2a_).

Figure 14a–d present the experimental results for case 10. This incident indicates an external fault, since the fault happens on the ‘*B*’ phase machine stator winding (between *B1* and *B10*). It is seen that the two currents (*i*_*1a*_ and *i*_*2a*_) are identical with no increase in their magnitudes, as presented in Fig. 14a. As demonstrated in Fig. 14b, the cross-coherence factor (*Ci*_*12a*_) is nearly 1.0. Consequently, there is no internal shunt fault on the ‘*A*’ phase motor stator winding. Also, it is obvious that the two auto-coherence factors (*Ci*_*1a*_*,* and *Ci*_*2a*_) are almost 1.0, as shown in Fig. 14d. Subsequently, there is no TTTF on the ‘*A*’ phase stator winding of the motor. In this laboratory experiment, the fault location is outside the machine protection zone. As a result, the protection algorithm does not send any tripping signal to the annunciation panel, as described in Fig. 14c. However, the fault incident is inside the protection region of the ‘*B*’ phase machine stator winding, which can be found using the developed ‘*B*’ phase differential relay based on the coherence function.

#### Case 11: TTTF (C1–C10)

**Fig. 15:**
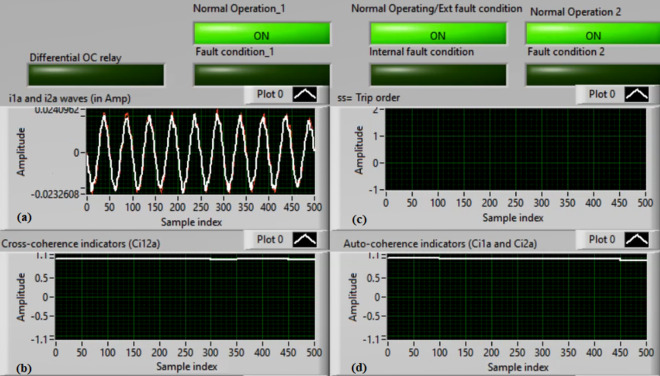
Results for case 11. (**a**) Two measured currents (i_1a_ and i_2a_), (**b**) Cross-coherence estimator (Ci1_2a_), and (**c**) Blocking signal, and (**d**) Auto-coherence estimators(Ci_1a_ and Ci_2a_).

Figure 15a–d present the experimental results for case 11. This scenario signifies an external fault because the fault occurs on the ‘*C*’ phase motor stator winding (between *C1* and *C10*). Figure 15a shows the two current signals (*i*_*1a*_ and *i*_*2a*_). Figure 15b depicts the cross-coherence estimator (*Ci*_*12a*_), and Fig. 15d illustrates the two auto-coherence estimators (*Ci*_*1a*_*,* and *Ci*_*2a*_). The three coherence estimators (*Ci*_*12a*_*, Ci*_*1a*_*,* and *Ci*_*2a*_) are approximately 1.0 during the display time. Thus, there is no internal shunt fault or turn-to-turn fault on the ‘*A*’ phase stator winding of the machine. In this circumstance, the fault location is outside the protection zone of the ‘*A*’ phase motor stator winding. As a result, there is no tripping signal, as shown in Fig. 15c. But the fault event is within the protection zone of the ‘*C*’ phase motor stator winding, which can be detected using the proposed ‘*C*’ phase differential relay that is contingent on the coherence algorithm.

#### Case 12: TTTF (B3–C3)

**Fig. 16:**
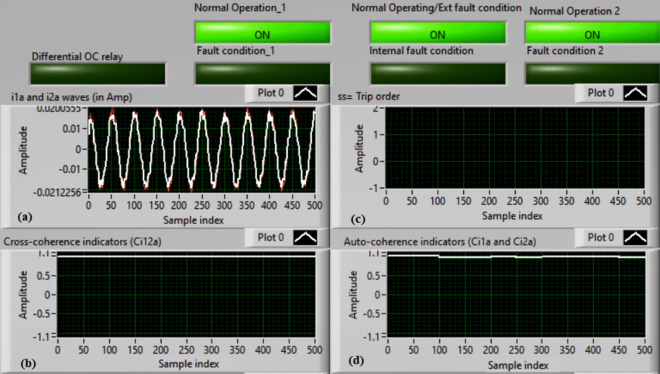
Results for case 12. (**a**) Two measured currents (i_1a_ and i_2a_), (**b**) Cross-coherence estimator (Ci1_2a_), and (**c**) Blocking signal, and (**d**) Auto-coherence estimators(Ci_1a_ and Ci_2a_).

Figures 16a–d show the experimental results for case 12. This scenario is an external shunt fault as it originates from *B3* to *C3*. Figure 16a introduces the two current waveforms (*i*_*1a*_ and *i*_*2a*_). Figure 16b illustrates the cross-coherence coefficient (*Ci*_*12a*_), and Fig. 16d depicts the two auto-coherence coefficients (*Ci*_*1a*_*,* and *Ci*_*2a*_). The three coherence coefficients (*Ci*_*12a*_*, Ci*_*1a*_*,* and *Ci*_*2a*_) are roughly 1.0 during the display time. Therefore, there is no internal shunt fault or turn-to-turn fault on the ‘*A*’ phase stator winding of the motor. In this case, the fault location is external with respect to the protection zone of the ‘*A*’ phase machine stator winding. As a consequence, there is not any tripping signal (emanating from the presented ‘*A*’ phase differential relay), as depicted in Fig. 16c. However, the fault event is within the protection zones of the ‘*B’* and ’*C*’ phase machine stator windings, which can be determined using the proposed ‘*B’* and ’*C*’ phase differential relays based on the coherence technique.

#### Case 13: TTTF (B2–C2)

**Fig. 17:**
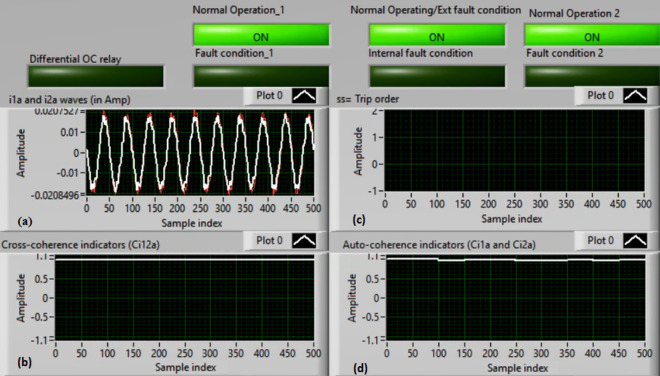
Results for case 13. (**a**) Two measured currents (i_1a_ and i_2a_), (**b**) Cross-coherence estimator (Ci1_2a_), and (**c**) Blocking signal, and (**d**) Auto-coherence estimators(Ci_1a_ and Ci_2a_).

Figures 17a–d introduce the experimental results for case 13. This incidence represents an external shunt fault as the fault is located between *B2* and *C2*. Figure 17a offers the two currents (*i*_*1a*_ and *i*_*2a*_). Figure 17b presents the cross-coherence coefficient (*Ci*_*12a*_), and Fig. 17d describes the two auto-coherence coefficients (*Ci*_*1a*_*,* and *Ci*_*2a*_). The three coherence estimators (*Ci*_*12a*_*, Ci*_*1a*_*,* and *Ci*_*2a*_) are nearly 1.0 during the display time. Thence, there is no internal shunt fault or turn-to-turn fault on the ‘*A*’ phase stator winding of the motor. In this test, the fault is external with respect to the protection region of the ‘*A*’ phase machine stator winding. As a result, there is no tripping signal (emanating from the presented ‘*A*’ phase differential relay), as observed in Fig. 17c. The practical results of Case 13 are similar to those of Case 12.

#### Case 14: TTTF (B10–C10)

**Fig. 18:**
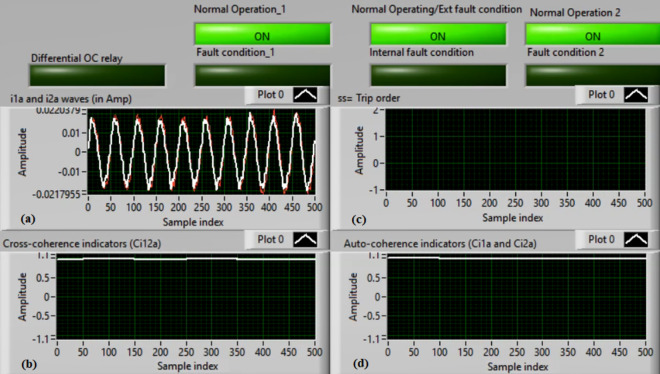
Results for case 14. (**a**) Two measured currents (i_1a_ and i_2a_), (**b**) Cross-coherence estimator (Ci1_2a_), and (**c**) Blocking signal, and (**d**) Auto-coherence estimators(Ci_1a_ and Ci_2a_).

Figures 18a–d reveal the experimental results for case 14. This situation indicates an external shunt fault because the fault is positioned between *B10* and *C10*. Figure 18a exhibits the two current curves (*i*_*1a*_ and *i*_*2a*_). Figure 18b depicts the cross-coherence measure (*Ci*_*12a*_), and Fig. 18d shows the two auto-coherence quantities (*Ci*_*1a*_*,* and *Ci*_*2a*_). The three coherence quantities (*Ci*_*12a*_*, Ci*_*1a*_*,* and *Ci*_*2a*_) are approximately 1.0 during the display time. Accordingly, there is no internal shunt fault or turn-to-turn fault situated on the ‘*A*’ phase stator winding of the motor. In this investigation, the fault situation is out of the protection zone of the ‘*A*’ phase machine stator winding. Consequently, there is no tripping flag (sent by the proposed ‘*A*’ phase differential relay), as illustrated in Fig. 18c. The algorithm results of Case 14 are similar to those of Case 13.

#### Case 15: Internal shunt fault (A2–B2)

**Fig. 19:**
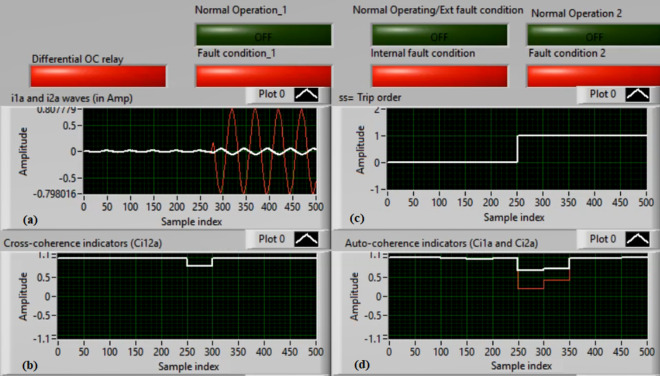
Results for case 15. (**a**) Two measured currents (i_1a_ and i_2a_), (**b**) Cross-coherence estimator (Ci1_2a_), and (**c**) Tripping signal, and (**d**) Auto-coherence estimators(Ci_1a_ and Ci_2a_).

Figures 19a–d show the experimental results for case 15. This scenario is internal shunt fault (*A2–B2*) because the fault occurs between the ‘*A*’ and ‘*B*’ phases of the motor stator windings. As shown in Fig. 19a, the two currents (*i*_*1a*_ and *i*_*2a*_) are unsymmetrical. As presented in Fig. 19b, the cross-coherence coefficient (*Ci*_*12a*_) is lower than the coherence setting of 0.95. Moreover, both the auto-coherence factors (*Ci*_*1a*_, and *Ci*_*2a*_) are unequal and less than the coherence setting value, as illustrated in Fig. 19d. Consequently, the recorded outcomes reveal that the internal shunt fault is present. The proposed ‘*A’* phase differential relay is able to identify the internal shunt fault located between the ‘*A*’ and ‘*B*’ phases of the machine stator windings. Furthermore, the proposed ‘*B’* phase differential relay can detect this fault classification. In this experiment, a tripping signal is announced by the proposed method, as presented in Fig. 19c.

#### Case 16: Internal shunt fault (A3–B3) through fault arc

**Fig. 20:**
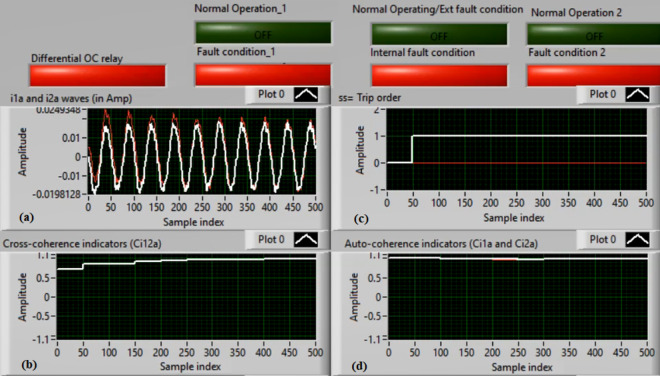
Results for case 16. (**a**) Two measured currents (i_1a_ and i_2a_), (**b**) Cross-coherence estimator (Ci1_2a_), and (**c**) Tripping signal, and (**d**) Auto-coherence estimators(Ci_1a_ and Ci_2a_).

Figures 20a–d show the experimental results for case 16. This occasion is internal shunt fault (*A3–B3*). As depicted in Fig. 20a, the two currents (*i*_*1a*_ and *i*_*2a*_) are dissimilar. As presented in Fig. 20b, the cross-coherence factor (*Ci*_*12a*_) is less than the coherence pickup value of 0.95. While, both auto-coherence coefficients (*Ci*_*1a*_*,* and *Ci*_*2a*_) are similar and approaching the coherence pickup value, as described in Fig. 20d. Subsequently, the experimental results prove that the internal shunt fault is existing. The proposed ‘*A’* phase differential relay has the ability to identify the internal shunt fault positioned between the ‘*A*’ and ‘*B*’ phases of the machine stator windings. Additionally, the developed ‘*B’* phase differential relay can identify this fault type. In this test, a tripping signal is issued to the annunciation platform by the proposed algorithm, as displayed in Fig. 20c.

Appendix (3) includes additional experimental results (spanning from case 17 to case 30) for the proposed protection scheme.

In summary, the above results confirm that the proposed approach based on the coherence criterion for the AC machine protection is capable of:

1. defining the normal operating conditions (as shown in case 1),
2. avoiding the impact of the motor starting current (as shown in case 2),
3. identifying the inter-turn faults (as shown in case 3 and cases 5–9),
4. finding the series faults (as shown in case 3 and cases 5–9) and the shunt faults (as shown in cases 15–16),
5. discriminating faults inside and outside the machine’s protection zone (external faults are shown in cases 10–14, and internal shunt faults are shown in cases 15–16),
6. distinguishing between the faulty operations (as shown in case 3, cases 5–9, and cases 15–16) and the healthy operations (as shown in case 1),
7. presenting a new proposal for relay tripping-characteristic curves based on the coherence estimators, which is created to distinguish between inter-turn, external and internal faults. These curves have the quadrilateral form (as shown in Fig. 1),
8. developing new coherence-inverse time curves that are used to quantify the tripping time in the case of inter-turn faults using the auto-coherence level (as shown in Fig. 2).
9. estimating the tripping time using the auto-coherence factor in the case of turn-to-turn faults (as given in Tables 3, 4).
10. specifying and estimating the imbalance level for the three-phase current waveforms, and
11. categorizing the ten internal shunt faults within the equipment protection zone (as shown in Fig. 3b–c),

**Table 4:** Estimated tripping times for the turn-to-turn faults (inside the machine protection zone-Phase *A*).

Case No	System fault type	*i*_*1a*_* ≈ i*_*2a*_ (*Amp*)	*Ci*_*1a*_* ≈ Ci*_*2a*_	Estimated tripping time *T*_*1op*_* ≈ T*_*2op*_ (in milliseconds)
			The selected coherence pickup is *C*_*pu*_ = *1.0* − *Δs*_*2*_ = *0.95*	
Case 3	TTTF (A1–A2) Phase A—Series Fault	0.084	0.21	284
Case 4	TTTF (A1–A2) Phase A—Series Fault	0.118	0.90	∞ The relay has failed to trip (i.e., Error action)
Case 5	TTTF (A1–A3) Phase A—Series Fault	0.182	0.18	234
Case 6	TTTF (A1–A4) Phase A—Series Fault	0.258	0.13	158
Case 7	TTTF (A1–A5) Phase A—Series Fault	0.343	012	145
Case 8	TTTF (A1–A7) Phase A—Series Fault	0.548	0.00	0
Case 9	TTTF (A1–A10) Phase A—Series Fault	0.677	0.10	118 The relay has sent a tripping action, but it is delayed time with compared to the previous case

## Algorithm effectiveness

### Algorithm performance estimation

In this study, the total number of trips was 1300, and 5.0 out of them were incorrect trips. The number of scenarios in which the algorithm failed to trip is 12.0. When the system’s operation was normal, the protection algorithm was constrained 70 times without any problems. Table 5 shows the procedures for estimating the accuracy, dependability, security, and reliability of the algorithm under the influence of various fault types (including turn-to-turn, winding-to-winding and winding-to-neutral faults, as well as the error of current transformers)^29,30^.

**Table 5:** Evaluation of the protection characteristics.

Power model condition	Number of scenarios	Malfunctioning times
The total number of trips	1300	5
The total number of normal operations	70	0
Assessment of algorithm characteristics	H_1_ = The total number of tests = 1370	Malfunctioning times = 5
	H_2_ = The total number of trips	= 1300
	H_3_ = The number of correct trips	= 1295
	H_4_ = The number of tripping failures	= 12
	H_5_ = The number of desirable trips	= 1295 + 12 = 1307
	H_6_ = The number of incorrect trips	= 1300– 1295 = 5
	H_7_ = H_5_ + H_6_	= 1307 + 5 = 1312
	$\% D= \frac{H3 }{H5}\times 100$	= $\frac{1295}{1307}$ $\times$ 100 = $99.08 \%$
	$\% S= \frac{H3}{H2}\times 100$	= $\frac{1295}{1300}\times$ 100 = $99.62 \%$
	$\% R= \frac{H3}{ H7}\times 100$	= $\frac{1295}{1312}\times$ 100 = $98.70 \%$
	$\% A= \frac{H1-H4-H6}{H1}\times 100$	= $\frac{1370-12-5 }{1370} \times$ 100 = 98.76%

D, dependability; S, security; R, reliability, and A, accuracy.

### Coherence algorithm advantages

The coherence estimator’s advantages (in terms of detection speed, fault identification accuracy, computational efficiency, reliability, and sensitivity verification) are described below.

#### Detection speed

- The pre-determined size of the data window used to calculate the coherence coefficients can be used to control the technique speed or the fault detection time. The data window can be set within one cycle of the power frequency. The smaller the data window size, the faster the relay speed, and the lower the relay accuracy.
- It is capable of simultaneously detecting and assessing fault and unbalanced events (Since the coherence coefficient is a measure of the association between two data sets of measurements, and it reveals the degree of synchrony between them). Therefore, it is considered a suitable tool for evaluating the association degree and for identifying abrupt changes in the two data sets at the same time.

#### Fault identification accuracy

- A group of novel tripping curves is developed based on the coherence coefficients calculated for the currents measurements, which can be used to activate the protection algorithm during the fault and unbalance periods to prevent the system from being damaged; whereas, it constrains the protection operation in the balanced and normal operating situations for the power network.
- The algorithm can easily select the appropriate setting for the coherence estimators to distinguish between the fault and normal operating conditions, as well as between the external and internal faults.
- A balance between the two incompatible requirements of speed and accuracy should be considered. This is because the speed and accuracy have an inverse relationship. High-speed systems tend to be less accurate for the simple reason that a high-speed system has a lower amount of information available at its disposal for making decisions.

#### Computational efficiency

(I) In order to improve the computational efficiency, the measurement errors should be avoided. In this work, the following necessities have been considered:
  1. The data window concept has been used for estimating the coherence estimators. From a signal processing perspective, the data window could be used to smooth out variance from short-term variations. As a result, the effects of decent ripples, temporary faults and DC offset components in the electrical measurements can be eliminated using the selected reasonable data window,
  2. The Data Acquisition Card (DAC) has been set to operate in differential mode,
  3. Proper grounding and ground bonding in the experimental setup has been established,
  4. Anti-aliasing concept has been used,
  5. The current transformers are the same type and have the same CTR. Moreover, they have a high accuracy class.
(II) To find and assess the current unbalance, the cross-coherence coefficients (estimated between each two phase currents) have taken into consideration the effects of both negative and zero sequence components. Furthermore, the coherence coefficient could detect a change in any power quality parameter of the electrical waveforms. Thus, the coherence statistic is considered a proper measure to evaluate the di-symmetry factor.

#### Algorithm reliability verification

The reliability of the protection is primarily defined by the absence of failures in the protective relay operation. The present algorithm can detect roughly all different types of shunt and series faults that cause variations in the machine currents. Thus, it is highly reliable. To ensure that the protection system will perform under larger-scale or more diverse real-world conditions, or to verify the reliability of the algorithm, several factors have been taken into consideration as follows:

1. Two current signals for each phase (or six current signals for the three-phase system) have been used as analog inputs to the protection algorithm. The aim of this study to observe changes in all signals at once,
2. The experimental setup has experienced extensive and different faults, including series and shunt faults,
3. Another experimental model has been used to examine the algorithm under different types of faults located on the AC machine windings, such as turn-to-turn, winding-to-neutral, and winding-to-winding,
4. A novel design for relay tripping curves has been established. The coherence-based tripping curves could be used to operate the protection algorithm during fault and imbalance events, preventing any system harm. In normal and balanced operation, the tripping curves hold the algorithm’s operation,
5. Accurate design, correct installation/testing, and appropriate elements are essential components of the entire system to ensure its reliability,
  For larger-scale power systems, the reliability of the protection system includes the reliability of various components, like AC input circuits, digital protective relays, and the operation of output circuits.
  AC input circuits: They include Current Transformers (CTs).
  The protective relays: They include the hardware and software of the relays.
  The output circuits: They include the connecting cables and circuit breaker that contains trip coils, moving contacts, and operating mechanism.
6. The reliability of the relaying system reliability can be achieved through redundancy (i.e., duplicating the relaying system). Our algorithm has two redundant algorithms. For larger-scale power systems, a backup protection can be used to improve the system’s reliability. However, the criticality of the power equipment affects the redundancy of the protection.
7. Various tests have been conducted to investigate the effectiveness of the protection algorithm as follows:
  (I) Simulation testing.
    The behavior of the AC machine under different fault scenarios can be modeled using the ATP simulation, and the MATLAB package is used to process the algorithm to examine the protection system. The coherence settings, logic, and coordination of the protection method can be verified through simulation testing. However, simulation testing has some limitations, such as the accuracy of the model, the difficulty of simulating complex faults, and the time required to run the simulation.
  (II) Primary testing
    Moreover, the protection system has been examined through primary testing, which enables the input of currents into the system and measures their responses. The primary testing requires the DAC, personal computer, and LABVIEW package. The primary testing can assure the functionality, accuracy, and sensitivity of the protection method, as well as their performance under real conditions. However, primary testing has some challenges, such as the safety of the participants and the availability of different power models.

#### Algorithm sensitivity verification

Power systems often deal with noisy environments due to various external and internal factors (e.g., harmonics, switching transients). Some precautions have been taken to avoid the effect of the measurement errors on the coherence estimators (due to high-frequency noise, harmonic distortions, or signal interference), as mentioned above in the point ‘(C) Computational efficiency’.

The sensitivity of the proposed protection is controllable using the coherence setting deviations and the size of data window.

#### Settings identification

- The coherence setting values and their deviations have been adjusted practically by watching the alarm flag and relay response signal at normal operating situation of the system using the LABVIEW software.
- The lower setting deviations, the lower relay accuracy. Also, the lower data window size, the larger relay speed and the lower relay accuracy. As mentioned before, a balance between the algorithm speed and accuracy should be considered.

#### Protection characteristics

In this study, the coherence coefficient settings can be employed to regulate the blocking and tripping zones located within the proposed relay characteristic. The following items explain how they affect the relay properties:

1. In the event of an overload current, the security can be enhanced by increasing both the data window size and the restraining zones located inside the proposed characteristics (using the coherence setting deviations). This is similar to increasing the pickup current of traditional differential current and overcurrent relays,
2. If the fault current is low, the sensitivity can be improved by reducing both the data window size and the restraining zones located inside the proposed characteristics (using the coherence setting deviations). This is the same as decreasing the pickup current of the traditional differential current and overcurrent relays,
3. The data window concept used to compute the coherence coefficients serves as an operating time delay and a digital low-pass filter for the analog input signals of the protective relay. This achieves stable operation for the protection system, and enables the power network to remain stable in certain conditions, such as CT linear errors, DC components, decent harmonics, and temporary/transient faults.
4. The proposed algorithm is characterized by high reliability due to its immunity to fault time duration, fault inception angle, fault location, and fault type. It is also able to detect all types of shunt and series faults, which cause current signals to vary in different working states of the AC machine.
5. The suggested protective relay satisfies the protection requirements of stability and reliability. The relay stability is determined by the efficiency of the method for identifying the electrical system’s operating conditions, while the relay reliability is primarily defined by the absence of failures in the operation of the protective relays.
6. A compromise in the relay settings is still necessary to ensure the coordination between the stability and dependability, the speed and accuracy, and the security and sensitivity. Thus, the coherence setting deviations have been selected to be 0.05, and the data window size has been taken one cycle in the proposed algorithm to balance these requirements.

### Critical comparison

In this section, there is direct comparison of the performance metrics (e.g., accuracy, detection time, false positives) with existing fault detection methods. Table 6 provides a comparison between the proposed protection methodology based on the coherence criterion and other recently published works.

**Table 6:** A comparison between the proposed methodology based on the coherence criterion and other recently published works.

References	Disadvantages	Advantages of the proposed methodology
References^29,30^	1. In^29^, the method was dependent on the mathematical models of the alienation statistic derived using the coherence calculated for the generator voltage and current measurements. As a result, the operating time of the alienation-based technique is higher than that of the coherence-based technique. Whereas, in^30^, the approach was contingent on the coherence statistic estimated for the measurements of the generator voltages and currents 2. In^29,30^, the unbalance factors were evaluated using cross-alienation and cross-coherence estimators, respectively, for the generator voltage and current measurements 3. In^29,30^, the protection schemes were unable to: - define the faults that occur between turns, - discriminate between external and internal faults in the machine protection zone, and - classify the ten internal shunt faults located within the equipment protection zone 4. Protection properties: - In^29^, the quantitative results revealed the following percentages for the protection properties: $\text{Dependability}$ (%) = $97.62\text{ \%}$ $\text{Security}$ (%) = $96.47\text{ \%}$ $\text{Reliability}$ (%) = $94.2 5\text{ \%}$ $\text{Accuracy}$ (%) = 95.83% - In^30^, the quantitative results demonstrated the following ratios for the protection characteristics: $\text{Dependability }(\text{\%})$ = $99.13\text{ \%}$ $\text{Security}$ (%) = $99.13\text{ \%}$ $\text{Reliability}$ (%) = $98.28\text{ \%}$ $\text{Accuracy}$ (%) = 98.52%	1. The proposed method has used the direct models of the coherence statistic computed for the machine current measurements. Therefore, the operating time of the coherence-based approach is shorter than that of the alienation-based approach 2. The unbalance factors have been directly evaluated using the cross-coherence estimators for the machine current measurements 3. The methodology is able to: - define the turn-to-turn faults, - discriminate between external and internal faults in the machine protection zone, and - classify the ten internal shunt faults placed within the equipment protection zone 4. Protection properties: - The protection properties of the suggested scheme are as follows: $\text{Dependability}$ (%) = $99.08\text{ \%}$ $\text{Security}$ (%) = $99.62\text{ \%}$ $\text{Reliability}$ (%) = $98.70\text{ \%}$ $\text{Accuracy}$ (%) = 98.76% - It is obvious that the present strategy is more secure, reliable, and accurate than the approaches in^29,30,36^ - It is clear that the suggested scheme is more dependable than the approaches in^29,36^ As a consequence, the assessment findings of the proposed solution demonstrate that it is superior to the existing techniques^29,30,36^ for protecting the stator windings of the AC machines
Reference^36^	- In^36^, the quantitative results illustrated the following ratios for the protection characteristics: $\text{Dependability}$ (%) = $94.44\text{ \%}$ $\text{Security}$ (%) = $97.14\text{ \%}$ $\text{Reliability}$ (%) = $91.89\text{ \%}$ $\text{Accuracy}$ (%) = 96.00%	
Reference^37^	1. In article^37^, a larger sampling rate was used for analyzing harmonic components. A delay in the operational time would be caused by the choice of 12,800 samples/Sec	1. The proposed methodology has been used 50 samples/ cycle (i.e., 2500 samples/Sec). As a result, it is faster than the method in^37^ 2. In the proposal, an execution time of one cycle is required for the imbalance assessment. Additionally, the operational time of the algorithm is controllable
Reference^38^	1. In^38^, the authors utilized the voltage imbalance model defined by the IEC, which relies on estimating the ratio of negative to positive symmetrical components. The method ignores the zero symmetrical components. Thus, the method in^38^ has a lower precision than the proposed technique 2. In^38^, The unbalance estimation used the RMS values of the three-phase voltage measurements 3. In^38^, the method used only the three-phase supply voltage signals	1. To identify and evaluate the imbalance situation, the cross-coherence estimators (calculated between each two phase currents) combine the effects of both zero and negative symmetrical components. Moreover, the coherence estimators can define a variation in any parameter of power quality (including wave shape, frequency, magnitude, phase displacement and symmetry). Therefore, the coherence criterion is a commendable instrument for estimating the imbalance coefficients 2. The unbalance measure is based on calculating the coherence estimators obtained for each data window. The data window size can be modified according to the protection requirements 3. The present approach has used the six current signals measured at the two sides of the machine stator windings
Reference 39	1. In^39^, different mathematical models were used. Therefore, the method used several threshold values, which were set spatially for diverse power quality phenomena in the power grid 2. In^39^, the method spent more time to get the compound index used to evaluate power quality phenomena	1. The advanced technique measures the imbalance severity utilizing the coherence method, and the imbalance assessment is swift since it spends about one cycle

## Algorithm limitations

To apply this algorithm to larger or more complex power systems, certain assumptions must be made, as follows:

(A) Firstly, there are some common requirements for all protection systems in order to ensure the proper operation of the proposed current relay. These requirements are as follows:
(1) The three-phase circuits connected to the protected equipment have the same type of Current Transformers (CTs), as well as the same accuracy class,
(2) Current Transformer Ratios (CTRs) are the same for the three-phase circuits, and
(3) Current measurements of the three-phase circuits are perfectly synchronized.
(B) Secondly, the primary limitations of the protection algorithm based on coherence coefficients are given below.
(1) Sample size affects coherence estimators.
  Small sample sizes can yield unreliable coherence estimates. When there are few data points, random fluctuations play a significant role. In other words, when analyzing coherence estimators, it is essential to consider a reasonable sample size. A small sample size may not yield sufficient data to accurately capture the correlation between variables. Conversely, a large sample size may lead to statistically significant results, even if the coherence coefficient is low. Therefore, it is imperative to take into account the sample size when interpreting the results of coherence estimators.
(2) The coherence estimator is insensitive to the scale and transformation.
(C) To mitigate the occurrence of incorrect tripping due to power quality problems, some precautions have been taken, as follows:
(1) A reasonable data window and suitable setting deviations has been selected,
(2) The Data Acquisition Card (DAC) has been set to run in differential mode,
(3) Proper grounding in the experimental setup has been established,
(4) Monitoring and analysis tools such as meters, sensors, and software have been used to measure and record the power quality parameters,
(5) The Nyquist sampling theorem stipulates that the sampling rate for the data acquisition system must be at least twice that of the maximum frequency of interest. The anti-aliasing concept has been applied in this work.
  In the proposed methodology, the selected sampling rate is 50 samples/cycle (or 2500 samples/Sec), which is greater than the minimum requirement of 10 times faster than the highest frequency of interest (10 × 50 Hz = 500 samples/Sec). Practically, the sampling frequency (in the LABVIEW software) is selected at 2500 Hz, which is greater than the minimum requirement of 2.5 times the frequency of interest = 2.5 × 50 Hz = 125 Hz.

## Main contributions

The main contributions in this investigation are listed below.

1. To distinguish between inter-turn, external and internal faults, a novel proposal of quadrilateral tripping curves based on cross-coherence and auto-coherence coefficients is developed.
2. The approach creates new coherence-time characteristic curves of the inverse type that can be used to determine the tripping time using the auto-coherence level in the case of turn-to-turn faults,
3. The protection functions of digital differential current and phase overcurrent relays can be combined into one protection scheme based on the coherence algorithm,
4. The method can classify the ten internal shunt faults within the machine protection zone using the cross-coherence criterion, and
5. The technique can be used to specify and assess the severity level of unbalance and disturbance for the three-phase current waveforms using the cross-coherence and auto-coherence estimators, respectively.

## Conclusions

In this article**,** the cross-coherence algorithm has been used to perform the functional role of the digital differential current protection to differentiate between the internal and external faults; whereas, the auto-coherence algorithm has been used to implement the function of the overcurrent relay to detect fault occurrences, such as internal, external, and turn-to-turn faults. The technique has been tested using a three-phase induction motor that has 20 taps for phase winding. This arrangement has been made to construct current transformers at the supply and neutral ends of the three-phase stator windings, and to facilitate executing comprehensive examinations to investigate the efficiency and efficacy of the developed approach. Numerous tests have been conducted for different fault scenarios, including but not limited to winding-to-winding, winding-to-neutral, and turn-to-turn faults. The testing results have revealed that the protection accuracy and reliability rates surpass 98.7%, and the protection security and dependability percentages overtake 99.0%. Experimentally, the coherence criterion has the ability to monitor diverse faults, distinguish between external and internal faults, detect turn-to-turn faults, and estimate the suitable tripping time for turn-to-turn faults. Besides, the sensitivity and time response of the fault detection are controllable. Moreover, a novel design of relay tripping curves based on the coherence coefficients has been established to distinguish between inter-turn, external and internal faults. In addition, new inverse coherence-time characteristics have been developed to identify the tripping time using the auto-coherence level in the case of turn-to-turn faults. Furthermore, the approach can classify the different internal shunt faults within the machine protection zone, and measure the intensity level of the three-phase currents unbalance and disturbance. Besides, it is able to protect the single-phase or three-phase stator winding(s) of the AC machines.

## Supplementary Information

Supplementary Information.

## Data Availability

All data generated or analysed during this study are included in this published article [and its supplementary information files].
