# Supplementary material for: Experimental performance examination of a coherence technique-based numerical differential current relay for AC machine stator windings protection
Source: Sci Rep. 2025 Mar 5;15:7751. doi: 10.1038/s41598-025-89092-z (PMC11882915; doi:10.1038/s41598-025-89092-z)
Supplement: Supplementary file 1 — Supplementary Information. [file 41598_2025_89092_MOESM1_ESM.pdf]

Nomenclatures:

Symbols	Abbreviations	Symbols	Abbreviations
CT	Current Transformer	$i_{1b}(n-N_c)$	The observation of the electrical current (i_{1b}) at the instant ($n-N_c$),
CTR	Current Transformer Ratio	$i_{2b}(n-N_c)$	The observation of the electrical current (i_{2b}) at the instant ($n-N_c$),
CB	Circuit Breaker	$i_{1c}(n-N_c)$	The observation of the electrical current (i_{1c}) at the instant ($n-N_c$),
DAC	Data Acquisition Card	$i_{2c}(n-N_c)$	The observation of the electrical current (i_{2c}) at the instant ($n-N_c$),
SLNF	Single-Line-to-Neutral Fault	*TTTFs*	Turn-to-Turn Faults
DLF	Double Line Fault	N_c	The number of observations per cycle of the fundamental power frequency for each current wave,
DLNF	Double Line-to-Neutral Fault	N_s	The number of observations per the data set ($N_s \leq N_c$) of the current wave,
3LNF	Three Line-to-Neutral Fault	Ci_{12x}	The cross-coherence estimator quantified between each two corresponding data sets for the two currents ($i_{1x}(n)$ and $i_{2x}(n)$) taken for '*X*' phase of the AC machine stator windings,
$i_{1a}(n)$, $i_{1b}(n)$ and $i_{1c}(n)$:	The observations of the three-phase currents at instant '*n*' measured at the three-phase supply end of the AC machine stator windings.	Ci_{1x}	The auto-coherence estimator quantified between each two consecutive data sets that are shifted from each other by one cycle for the electrical current $i_{1x}(n)$ acquired at the supply end,
$i_{2a}(n)$, $i_{2b}(n)$ and $i_{2c}(n)$:	The observations of the three-phase currents at instant '*n*' measured at the three-phase neutral end of the AC machine stator windings.	Ci_{2x}	The auto-coherence estimator quantified between each two consecutive data sets that are shifted from each other by one cycle for the electrical current $i_{2x}(n)$ acquired at the neutral end,
$i_{1a}(n-N_c)$	The observation of the electrical current (i_{1a}) at the instant ($n-N_c$),	*IM*	Induction machine,
$i_{2a}(n-N_c)$	The observation of the electrical current (i_{2a}) at the instant ($n-N_c$),	K_s	The selected time multiplier,
X	The subscript *X* is the phase designation *A, B,* or *C*.	C_{pu}	The coherence pickup of the algorithm (it is $C_{pu}= 1.0 - \Delta s_2= 0.95$),
T_{1op}	The operating time (in Sec) estimated using the auto-coherence (Ci_{1x}),	Δs_1, and Δs_2	The coherence setting deviations,
T_{2op}	The operating time (in Sec) estimated using the auto-coherence (Ci_{2x}),	Δs_1	the specified setting of the cross-coherence coefficient (Ci_{12x}), which is used to detect internal shunt faults,
Δs_2	The specified setting of the auto-coherence coefficient (Ci_{1x} or Ci_{2x}), which is used to define all kinds of fault,	Ci_{1xy}	The cross-coherence estimator quantified between each two corresponding data sets for the two currents ($i_{1x}(n)$ and $i_{1y}(n)$) taken for '*X* and *Y*' phases of the AC machine stator windings,
X and *Y*	Two different phases of the three phases *A, B,* and *C* are represented by the subscripts *X* and *Y*.	Ci_{2xy}	The cross-coherence estimator quantified between each two corresponding data sets for the two currents ($i_{2x}(n)$ and $i_{2y}(n)$) taken for '*X* and *Y*' phases of the AC machine stator windings,

Appendix (1): The specifications of the power model elements

The specifications of the power system elements	Numerical value
**Three-phase power supply:**	
Rated line voltage	*380 V*
Rated frequency	*50 Hz*
**Three-phase induction motor (under test):**	
Rated power	*2.9 kW (Star connection)*
Rated line voltage	*400 V*
Nominal frequency	*50 Hz*
Rated line current	*6.3 A*
Rated speed	*1415 rpm*
No. of taps per each winding	*20 Tapes*
No. of turns per each tap	*5 Turns*
Turn dimension	*0.8* mm^2
**Current transformers (CTs):**	
Current Transformer turns' Ratio (CTR)	*200/5*
Frequency	*47…50…63 Hz*
CT accuracy class	*1.0*
Rated burden	*2.5 VA*
CT burden	*1 Ω*
**Voltage Transformers (VTs)**	
Voltage Transformer turns' Ratio (VTR)	*220 / 6/ 3*
VT accuracy class	*0.5*
Nominal frequency	*50/60 Hz*
Rated burden	*25 VA*
**Miniature Circuit Breaker (MCB1)**	
Phase type	Three phase
Rated current	63 A
Rated voltage	400 V

Appendix (2): Input quantities of the proposed protection algorithm

**Quantity designation**	**Quantity description**	**Value**
$\boldsymbol{i_{1a}(n)}$, $\boldsymbol{i_{1b}(n)}$ **and** $\boldsymbol{i_{1c}(n)}$	The current measurements of *a*, *b* and *c* phases, respectively, at the instant *n* taken at the supply end of the AC machine stator windings,	They are measured on-line and updated
$\boldsymbol{I_{2a}(n)}$, $\boldsymbol{i_{2b}(n)}$ **and** $\boldsymbol{i_{2c}(n)}$	The current measurements of *a*, *b* and *c* phases, respectively, at the instant *n* taken at the neutral end of the AC machine stator windings,	
$\boldsymbol{F_c}$	The fundamental cycle frequency for electrical signals	50 *Hz*
$\boldsymbol{T_c}$	The cycle time interval	20 milliseconds
$\boldsymbol{F_{sp}}$	The frequency rate of the digital system	2.5 *kHz*
$\boldsymbol{T_{sp}}$	The sampling time span	0.4 milliseconds
$\boldsymbol{N_c}$	The sample size per each one cycle, $N_c = T_c / T_{sp}$ or $N_c = F_{sp} / F_c$	50 samples/cycle
$\boldsymbol{N_s}$	The sample size per each data set area, $N_s = N_c$	50 samples/data set
$\boldsymbol{T_{ds}}$	The full display time	10 cycles
$\boldsymbol{\Delta s_1}$	The predetermined deviation for the cross-coherence coefficients: (Ci_{12a}, Ci_{12b} and Ci_{12c})	+0.05
$\boldsymbol{\Delta s_2}$	The predetermined deviation for the auto-coherence coefficients: (Ci_{1a}, Ci_{1b}, Ci_{1c}, Ci_{2a}, Ci_{2b} and Ci_{2c})	+0.05

Appendix (3): Additional experimental results (from case 17 to case 30) for the proposed protection scheme

4.17 Case 17: Internal Shunt Fault (A3-B3)

Figs. S1(a-d) show the experimental results for case 17. Case 17 is internal shunt fault (*A3-B3*).

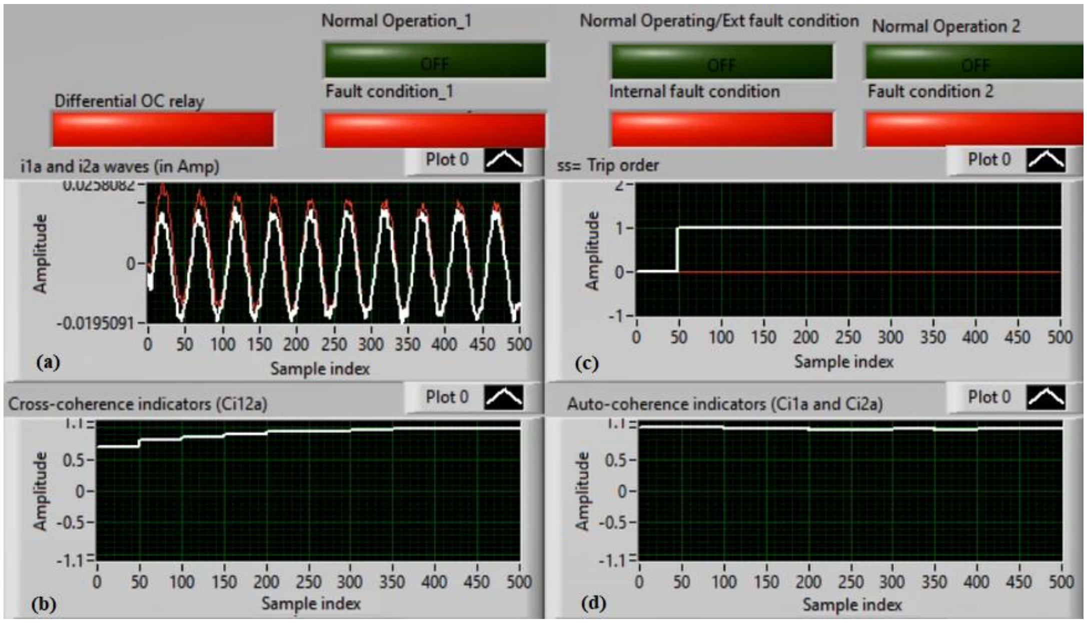

Fig. S1 Results for case 17

(a) Two measured currents (i_{1a} and i_{2a}), (b) Cross-coherence estimator (Ci_{12a}), and (c) Tripping signal, and (d) Auto-coherence estimators (Ci_{1a} and Ci_{2a}).

4.18 Case 18: Internal Shunt Fault (A5-B5)

Figs. S2(a-d) illustrate the experimental results for case 18. Case 18 is internal shunt fault (*A5-B5*).

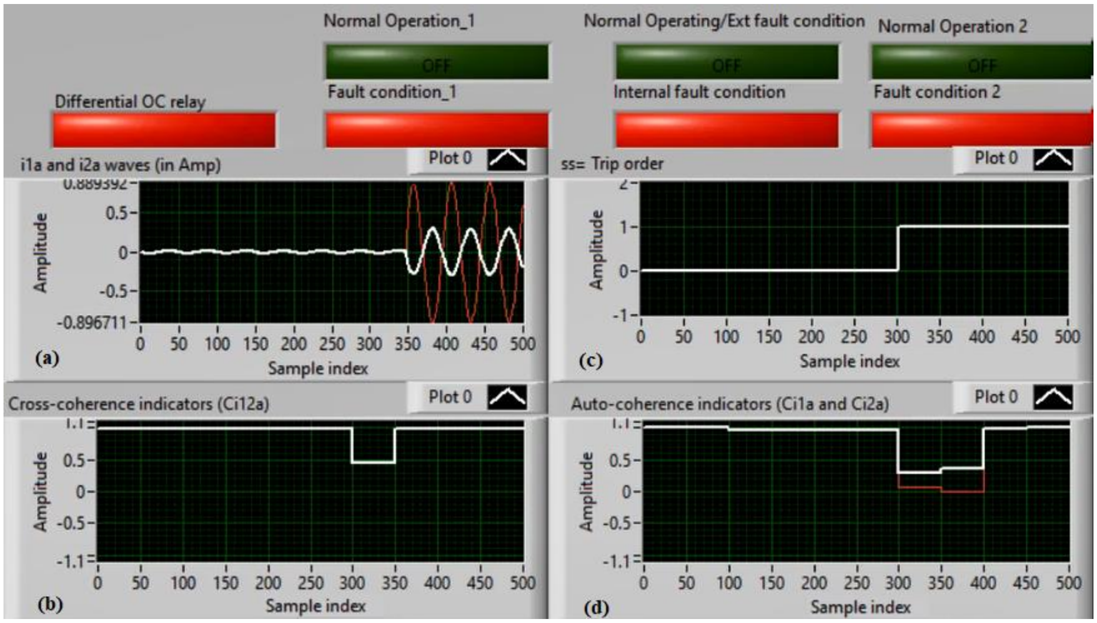

Fig. S2 Results for case 18

(a) Two measured currents (i_{1a} and i_{2a}), (b) Cross-coherence estimator (Ci_{12a}), and (c) Tripping signal, and (d) Auto-coherence estimators (Ci_{1a} and Ci_{2a}).

4.19 Case 19: Internal Shunt Fault (A10-B10)

Figs. S3(a-d) illustrate the experimental results for case 19. Case 19 is internal shunt fault (*A10-B10*).

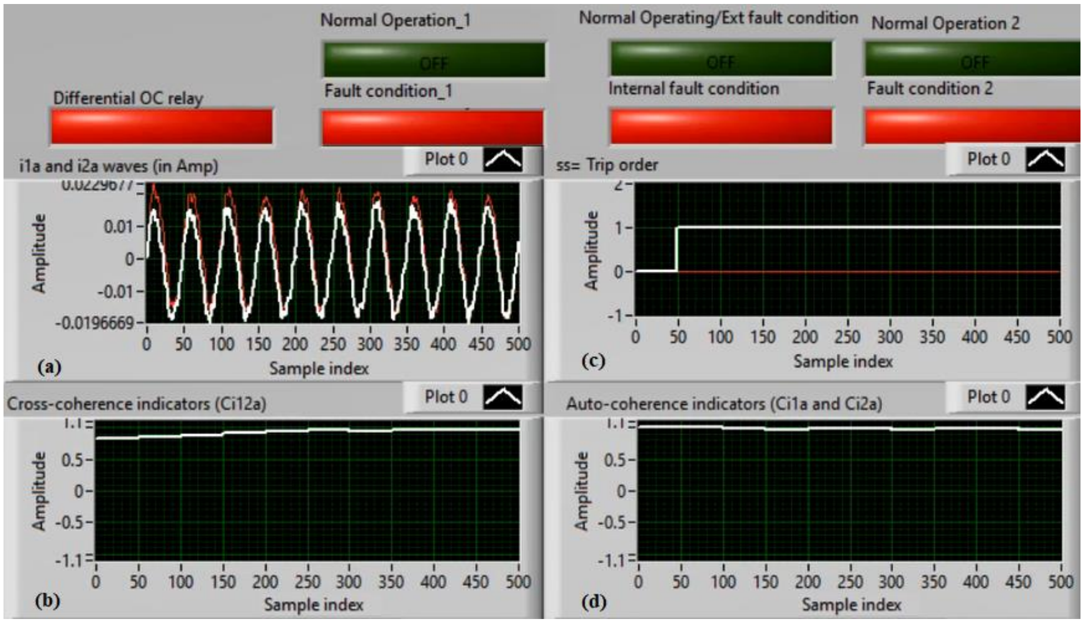

Fig. S3 Results for case 19

(a) Two measured currents (i_{1a} and i_{2a}), (b) Cross-coherence estimator (Ci_{12a}), and (c) Tripping signal, and (d) Auto-coherence estimators (Ci_{1a} and Ci_{2a}).

4.20 Case 20: Internal Shunt Fault (A10-B10)

Figs. S4(a-d) illustrate the experimental results for case 20. Case 20 is internal shunt fault (*A10-B10*).

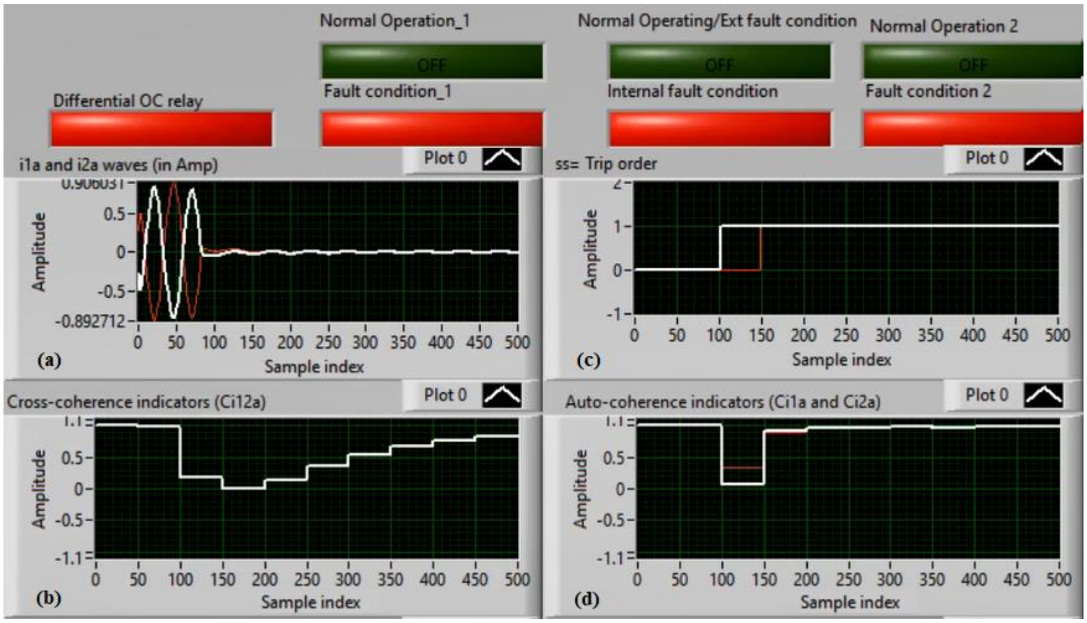

Fig. S4 Results for case 20

(a) Two measured currents (i_{1a} and i_{2a}), (b) Cross-coherence estimator (Ci_{12a}), and (c) Tripping signal, and (d) Auto-coherence estimators (Ci_{1a} and Ci_{2a}).

4.21 Case 21: Internal Shunt Fault (A3-C3)

Figs. S5(a-d) present the experimental results for case 21. Case 21 is internal shunt fault (*A3-C3*).

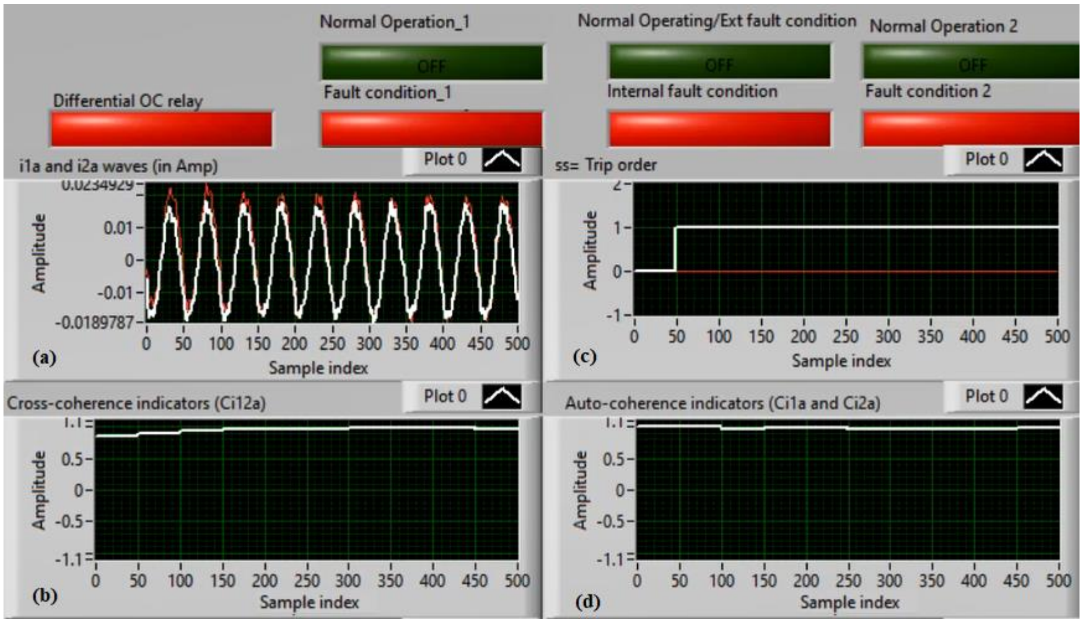

Fig. S5 Results for case 21

(a) Two measured currents (i_{1a} and i_{2a}), (b) Cross-coherence estimator (Ci_{12a}), and (c) Tripping signal, and (d) Auto-coherence estimators (Ci_{1a} and Ci_{2a}).

4.22 Case 22: Internal Shunt Fault (A3-C3)

Figs. S6(a-d) illustrate the experimental results for case 22. Case 22 is internal shunt fault (*A3-C3*).

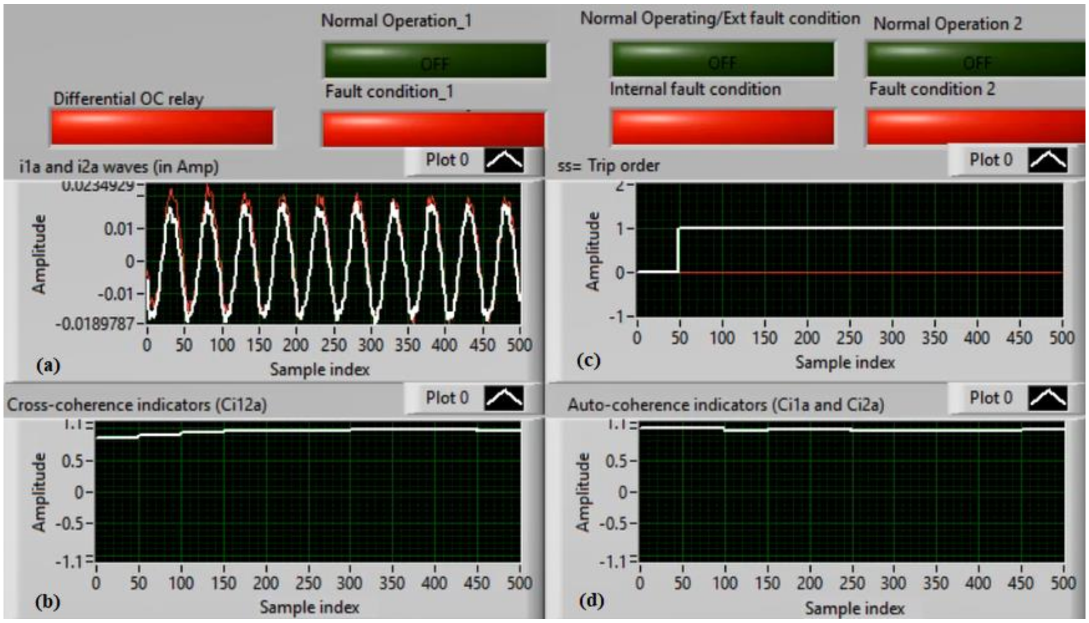

Fig. S6 Results for case 22

(a) Two measured currents (i_{1a} and i_{2a}), (b) Cross-coherence estimator (Ci_{12a}), and (c) Tripping signal, and (d) Auto-coherence estimators (Ci_{1a} and Ci_{2a}).

4.23 Case 23: Internal Shunt Fault (A5-C5)

Figs. S7(a-d) depict the experimental results for case 23. Case 23 is internal shunt fault (*A5-C5*).

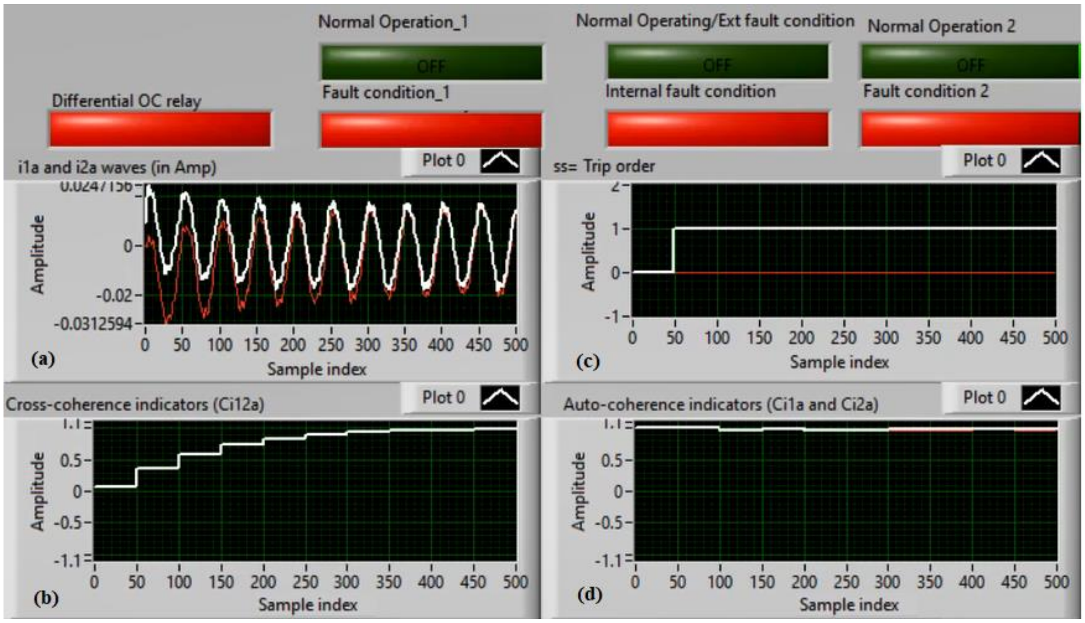

Fig. S7 Results for case 23

(a) Two measured currents (i_{1a} and i_{2a}), (b) Cross-coherence estimator (Ci_{12a}), and (c) Tripping signal, and (d) Auto-coherence estimators (Ci_{1a} and Ci_{2a}).

4.24 Case 24: Internal Shunt Fault (A6-C6)

Figs. S8(a-d) illustrate the experimental results for case 24. Case 24 is internal shunt fault (*A6-C6*).

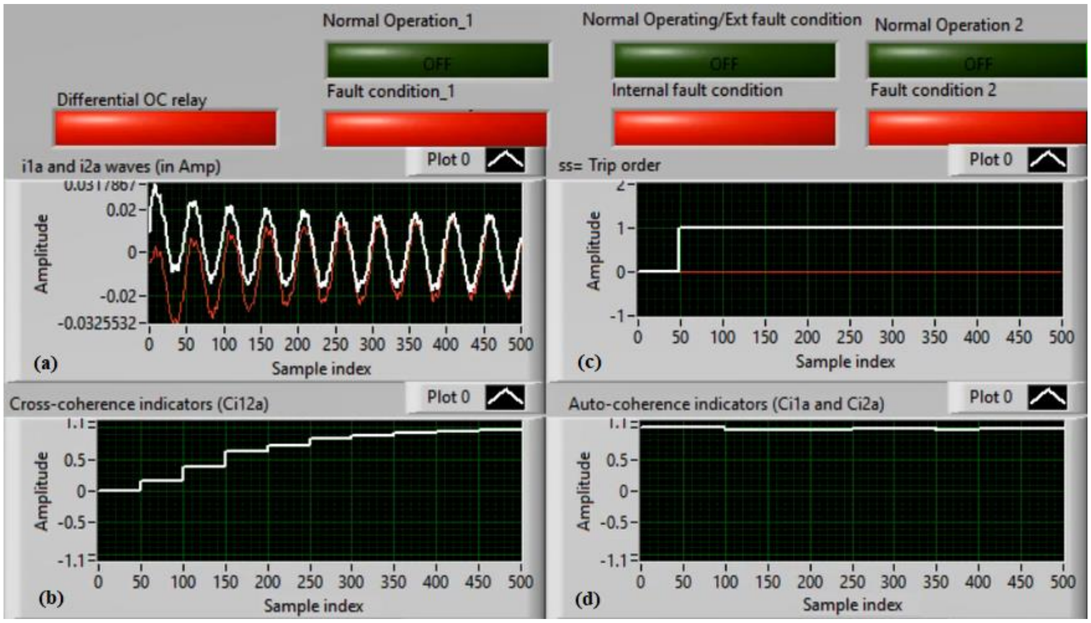

Fig. S8 Results for case 24

(a) Two measured currents (i_{1a} and i_{2a}), (b) Cross-coherence estimator (Ci_{12a}), and (c) Tripping signal, and (d) Auto-coherence estimators (Ci_{1a} and Ci_{2a}).

4.25 Case 25: Internal Shunt Fault (A10-C10)

Figs. S9(a-d) illustrate the experimental results for case 25. Case 25 is internal shunt fault (*A10-C10*).

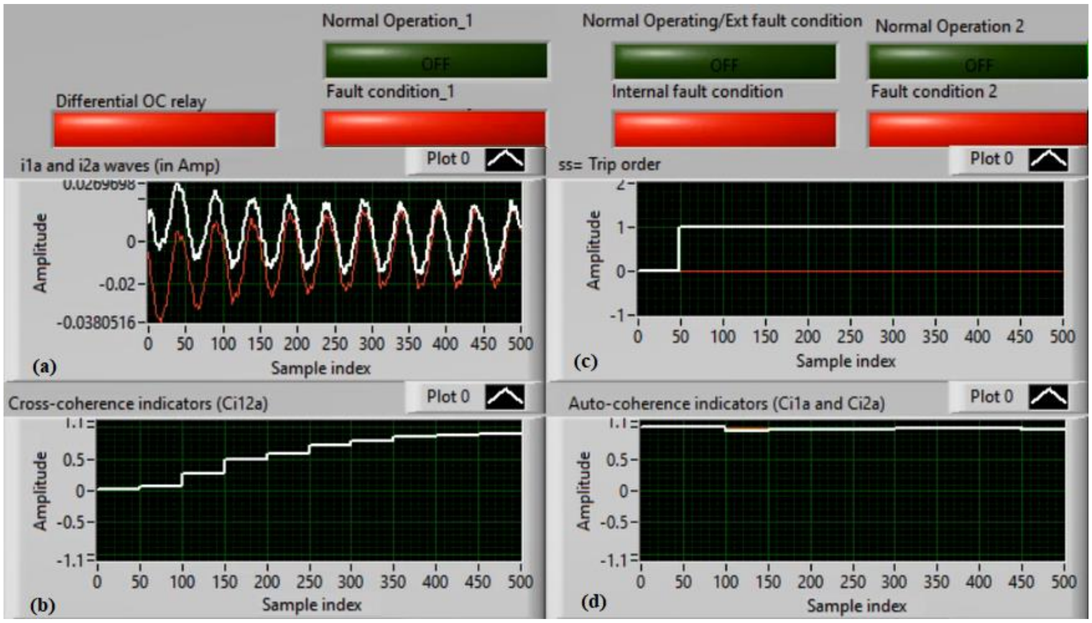

Fig. S9 Results for case 25

(a) Two measured currents (i_{1a} and i_{2a}), (b) Cross-coherence estimator (Ci_{12a}), and (c) Tripping signal, and (d) Auto-coherence estimators (Ci_{1a} and Ci_{2a}).

4.26 Case 26: Internal Shunt Fault (A6-B6)

Figs. S10(a-d) illustrate the experimental results for case 26. Case 26 is internal shunt fault (*A6-B6*).

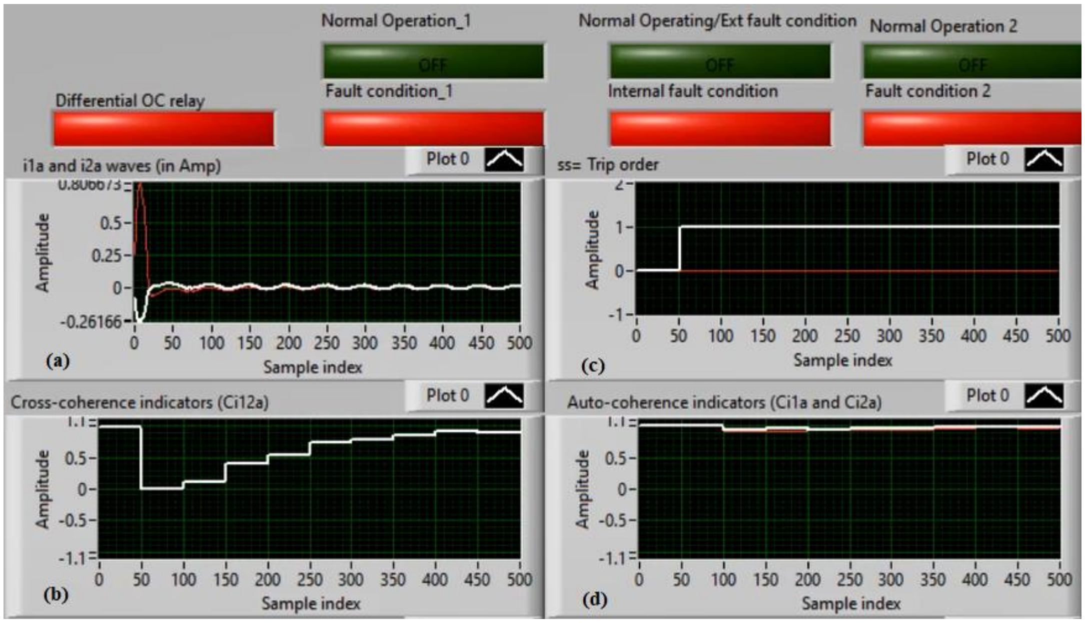

Fig. S10 Results for case 26

(a) Two measured currents (i_{1a} and i_{2a}), (b) Cross-coherence estimator (Ci_{12a}), and (c) Tripping signal, and (d) Auto-coherence estimators (Ci_{1a} and Ci_{2a}).

4.27 Case 27: Internal Shunt Fault (A4-B4)

Figs. S11(a-d) illustrate the experimental results for case 27. Case 27 is internal shunt fault (*A4-B4*).

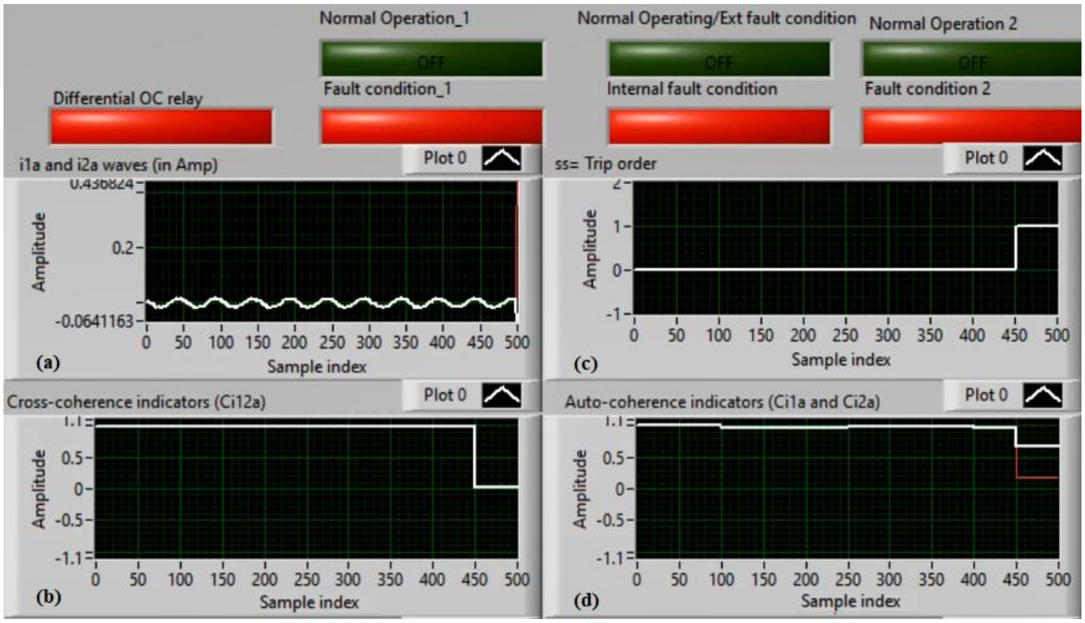

Fig. S11 Results for case 27

(a) Two measured currents (i_{1a} and i_{2a}), (b) Cross-coherence estimator (Ci_{12a}), and (c) Tripping signal, and (d) Auto-coherence estimators (Ci_{1a} and Ci_{2a}).

4.28 Case 28: Internal Shunt Fault (A4-B4)

Figs. S12(a-d) show the experimental results for case 28. Case 28 is internal shunt fault (*A4-B4*).

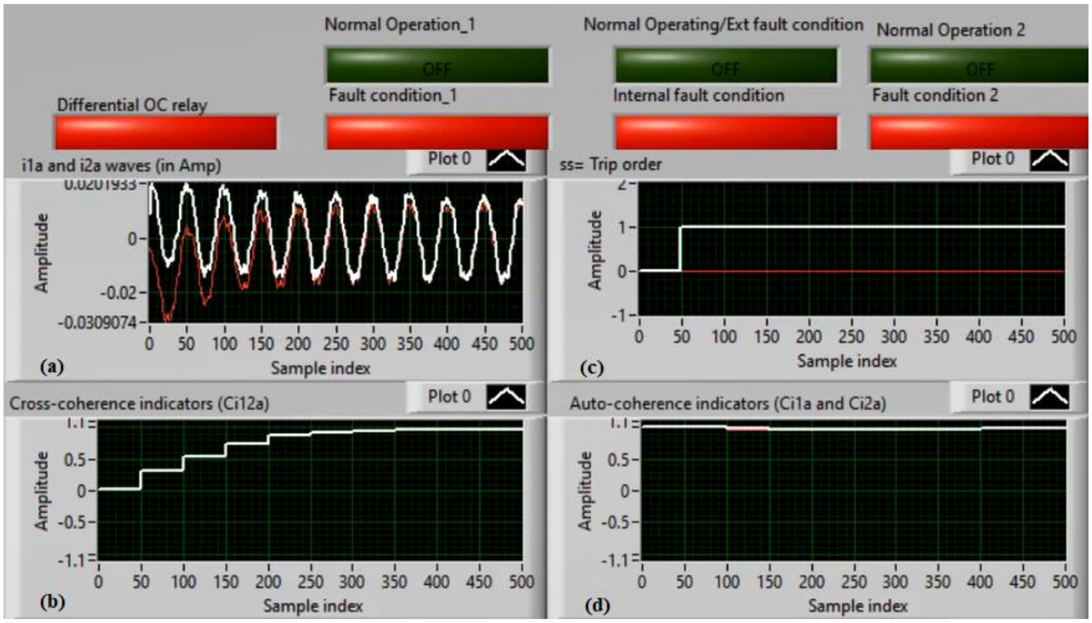

Fig. S12 Results for case 28

(a) Two measured currents (i_{1a} and i_{2a}), (b) Cross-coherence estimator (Ci_{12a}), and (c) Tripping signal, and (d) Auto-coherence estimators (Ci_{1a} and Ci_{2a}).

4.29 Case 29: Internal Shunt Fault (A10-B10)

Figs. S13(a-d) illustrate the experimental results for case 29. Case 29 is internal shunt fault (*A10-B10*).

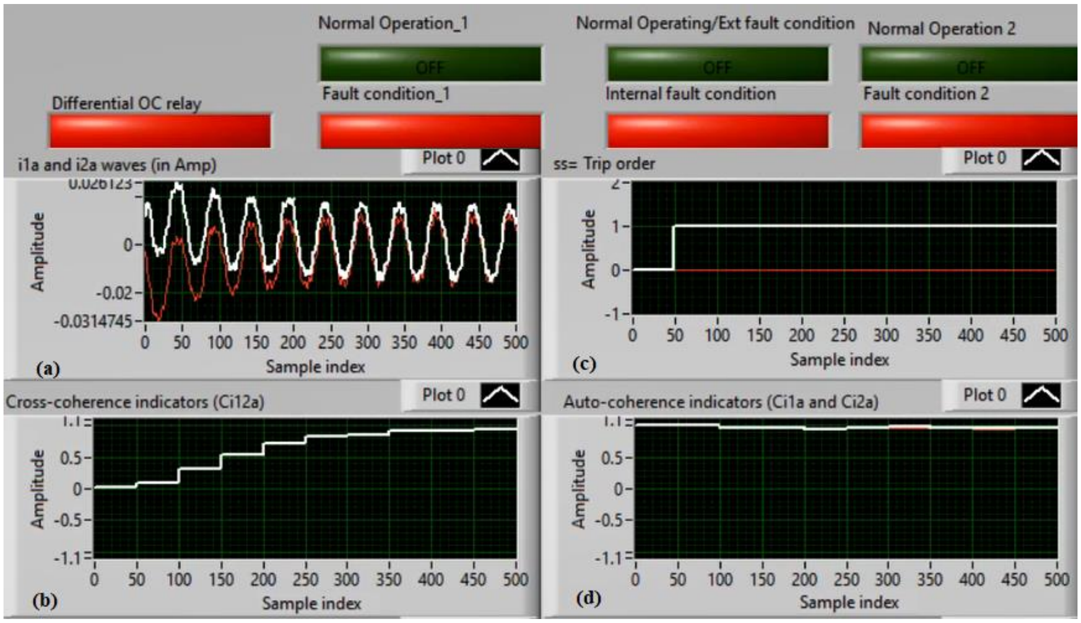

Fig. S13 Results for case 29

(a) Two measured currents (i_{1a} and i_{2a}), (b) Cross-coherence estimator (Ci_{12a}), and (c) Tripping signal, and (d) Auto-coherence estimators (Ci_{1a} and Ci_{2a}).

4.30 Case 30: Internal Shunt Fault (A4-B4)

Figs. S14(a-d) present the experimental results for case 30. Case 30 is internal shunt fault (*A4-B4*).

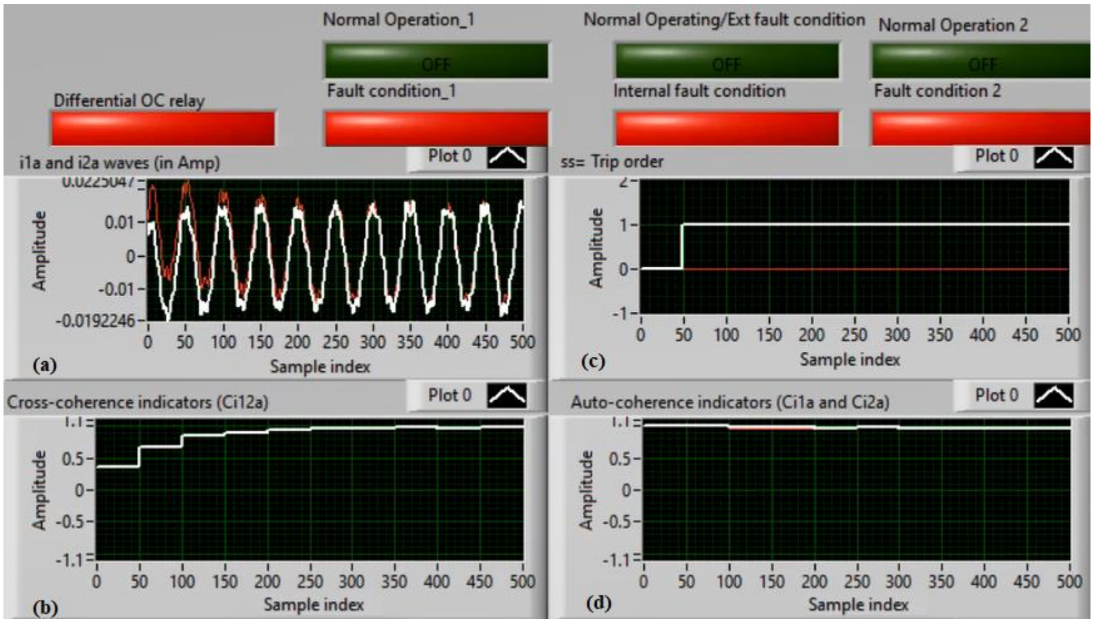

Fig. S14 Results for case 30

(a) Two measured currents (i_{1a} and i_{2a}), (b) Cross-coherence estimator (Ci_{12a}), and (c) Tripping signal, and (d) Auto-coherence estimators (Ci_{1a} and Ci_{2a}).
